# Research on Implementation of Interventions in Tuberculosis Control in Low- and Middle-Income Countries: A Systematic Review

**DOI:** 10.1371/journal.pmed.1001358

**Published:** 2012-12-18

**Authors:** Frank Cobelens, Sanne van Kampen, Eleanor Ochodo, Rifat Atun, Christian Lienhardt

**Affiliations:** 1 Department of Global Health, Academic Medical Center, Amsterdam, The Netherlands; 2 Amsterdam Institute of Global Health and Development, Amsterdam, The Netherlands; 3 KNCV Tuberculosis Foundation, The Hague, The Netherlands; 4 Department of Clinical Epidemiology, Biostatistics and Bioinformatics, Academic Medical Center, Amsterdam, The Netherlands; 5 Imperial College London, London, United Kingdom; 6 Stop TB Partnership, World Health Organization (WHO), Geneva, Switzerland; Universidad Peruana Cayetano Heredia, Peru

## Abstract

**Background:**

Several interventions for tuberculosis (TB) control have been recommended by the World Health Organization (WHO) over the past decade. These include isoniazid preventive therapy (IPT) for HIV-infected individuals and household contacts of infectious TB patients, diagnostic algorithms for rule-in or rule-out of smear-negative pulmonary TB, and programmatic treatment for multidrug-resistant TB. There is no systematically collected data on the type of evidence that is publicly available to guide the scale-up of these interventions in low- and middle-income countries. We investigated the availability of published evidence on their effectiveness, delivery, and cost-effectiveness that policy makers need for scaling-up these interventions at country level.

**Methods and Findings:**

PubMed, Web of Science, EMBASE, and several regional databases were searched for studies published from 1 January 1990 through 31 March 2012 that assessed health outcomes, delivery aspects, or cost-effectiveness for any of these interventions in low- or middle-income countries. Selected studies were evaluated for their objective(s), design, geographical and institutional setting, and generalizability. Studies reporting health outcomes were categorized as primarily addressing efficacy or effectiveness of the intervention. These criteria were used to draw landscapes of published research. We identified 59 studies on IPT in HIV infection, 14 on IPT in household contacts, 44 on rule-in diagnosis, 19 on rule-out diagnosis, and 72 on second-line treatment. Comparative effectiveness studies were relatively few (*n* = 9) and limited to South America and sub-Saharan Africa for IPT in HIV-infection, absent for IPT in household contacts, and rare for second-line treatment (*n* = 3). Evaluations of diagnostic and screening algorithms were more frequent (*n* = 19) but geographically clustered and mainly of non-comparative design. Fifty-four studies evaluated ways of delivering these interventions, and nine addressed their cost-effectiveness.

**Conclusions:**

There are substantial gaps in published evidence for scale-up for five WHO-recommended TB interventions settings at country level, which for many countries possibly precludes program-wide implementation of these interventions. There is a strong need for rigorous operational research studies to be carried out in programmatic settings to inform on best use of existing and new interventions in TB control.

*Please see later in the article for the Editors' Summary*

## Abstract 2

Cobelens and colleagues systematically reviewed research on implementation and cost-effectiveness of the WHO-recommended interventions for tuberculosis.

## Editors' Summary

**Background:**

Tuberculosis (TB), caused by *Mycobacterium tuberculosis*, is curable and preventable, but according to the World Health Organization (WHO), in 2011, 8.7 million people had symptoms of TB (usually a productive cough and fever) and 1.4 million people—95% from low- and middle-income countries—died from TB. TB is also the leading cause of death in people with HIV worldwide, and in 2010 about 10 million children were orphaned as a result of their parents dying from TB. To help reduce the considerable global burden of TB, a global initiative called the Stop TB Partnership, led by WHO, has implemented a strategy to reduce deaths from TB by 50% by 2015—even greater than the target of Millennium Development Goal 6 (to reverse the increase in TB incidence by 2015).

**Why Was This Study Done?:**

Over the past few years, WHO has recommended that countries implement several interventions to help control the spread of tuberculosis through measures to improve prevention, diagnosis, and treatment. Five such interventions currently recommended by WHO are: treatment with isoniazid to prevent TB among people who are HIV positive, and also among household contacts of people infected with TB; the use of clinical pathways (algorithms) for diagnosing TB in people accessing health care who have a negative smear test—the most commonly used diagnostic test, which relies on sputum samples—(“rule-in algorithms”); screening algorithms for excluding TB in people who have HIV (“rule-out algorithms”); and finally, provision of second-line treatment for multidrug-resistant tuberculosis (a form of TB that does not respond to the most commonly used drugs) under programmatic conditions. The effectiveness of these interventions, their costs, and the practicalities of implementation are all important information for countries seeking to control TB following the WHO guidelines, but little is known about the availability of this information. Therefore, in this study the researchers systematically reviewed published studies to find evidence of the effectiveness of each of these interventions when implemented in routine practice, and also for additional information on the setting and conditions of implemented interventions, which might be useful to other countries.

**What Did the Researchers Do and Find?:**

Using a specific search strategy, the researchers comprehensively searched through several key databases of publications, including regional databases, to identify 208 (out of 11,489 found initially) suitable research papers published between January 1990 and March 2012. For included studies, the researchers also noted the geographical location and setting and the type and design of study.

Of the 208 included studies, 59 focused on isoniazid prevention therapy in HIV infection, and only 14 on isoniazid prevention therapy for household contacts. There were 44 studies on “rule-in” clinical diagnosis, 19 on “rule-out” clinical diagnosis, and 72 studies on second-line treatment for TB. Studies on each intervention had some weaknesses, and overall, researchers found that there were very few real-world studies reporting on the effectiveness of interventions in program settings (rather than under optimal conditions in research settings). Few studies evaluated the methods used to implement the intervention or addressed delivery and operational issues (such as adherence to treatment), and there were limited economic evaluations of the recommended interventions. Furthermore, the researchers found that in general, the South Asian region was poorly represented.

**What Do These Findings Mean?:**

These findings suggest that there is limited evidence on effectiveness, delivery, and cost-effectiveness to guide the scale-up of five WHO recommended interventions to control tuberculosis in the countries and settings, despite the urgent need for such interventions to be implemented. The poor evidence base identified in this review highlights the tension between the decision to adopt the recommendation and its implementation adapted to local circumstances, and may be an important reason as to why these interventions are not implemented in many countries. This study also suggests creative thinking is necessary to address the gaps between WHO recommendations and global health policy on new interventions and their real-world implementation in country-wide TB control programs. Future research should focus more on operational studies, the results of which should be made publicly available, and researchers, donors, and medical journals could perhaps re-consider their priorities to help bridge the knowledge gap identified in this study.

**Additional Information:**

Please access these Web sites via the online version of this summary at http://dx.doi.org/10.1371/journal.pmed.1001358.

- WHO has a wide range of information about TB and research on TB, including more about the STOP TB strategy and the STOP TB Partnership
- The UN website has more information about MDG 6
- The Global Fund to Fight AIDS, Tuberculosis and Malaria has specific information about progress on TB control

## Introduction

Despite a widely adopted global strategy to control the disease, tuberculosis (TB) remains a major health problem, particularly in resource-poor countries [1]. New interventions are needed to improve diagnosis, treatment, and prevention of infection and disease, such as new technologies (e.g., diagnostic) or products (e.g., drugs, vaccines), but also novel use of existing technologies and products (e.g., alternative diagnostic algorithms, novel ways of improving treatment adherence) [2],[3]. Policy makers need to consider how new interventions can be adopted by TB control programs and implemented at a program-wide scale. This evaluation involves policy choices at several levels—global, national, and local—that need to be informed by evidence that has been collected and interpreted in a systematic and reproducible way [4], especially evidence on intervention effectiveness. While clinical trials (e.g., of new drugs) or laboratory-based comparisons against gold-standard methods (e.g., of new diagnostics) lay the basis for such evidence by establishing the efficacy under optimally controlled conditions, the effectiveness of an intervention quantifies effects in real-life health care settings [5], as measured by outcomes that are relevant to TB control (e.g., the number of patients cured, or the number of TB cases prevented) as well as outcomes that are relevant to patients (e.g., earlier diagnosis and cure, improved access to care) [4],[6]. In addition, policy makers need to understand how an efficacious intervention can best be delivered in various contexts, in particular the conditions and requirements that determine implementation success or failure. Such conditions may include, for example, methods for optimizing treatment adherence, or for assuring access to diagnostic procedures [7]–[9]. Finally, policy makers in resource-constrained settings need to know whether an intervention is the most cost-effective way of improving TB control compared to alternative interventions [10]. Collectively, these three types of information can provide the “evidence for scale-up,” addressing whether and how a new intervention will improve TB control in a cost-effective way at program-wide scale.

Over the past decade, several new interventions in TB control have been developed and recommended in World Health Organization (WHO) guidelines [11]. While recommendations for new interventions are usually based on evidence for efficacy arising from controlled studies, little is known about their effectiveness, the requirements for optimum delivery, and their cost-effectiveness when implemented in various epidemiological and resource conditions. This limited evidence, along with other factors, might have hindered wide-scale use of potentially effective interventions [12]. There are currently no systematically collected data on the availability of evidence for scale-up of newly recommended interventions for TB control. Therefore, we conducted a systematic review of published study reports of five related interventions that have been recommended by the WHO over the past decade, and for which evidence guiding scale-up would be needed. The five interventions were selected as representing direct actions to be carried out at the country level to improve TB control with regard to prevention, diagnosis, and treatment and covering a large spectrum of situations. This made it possible to assess what research had been done to guide implementation under a variety of epidemiological conditions (e.g., high or low HIV incidence, high or low burden of TB drug resistance).

These interventions are: (i) isoniazid preventive therapy (IPT) for preventing TB disease among HIV-infected individuals; (ii) IPT for preventing TB disease among household contacts of infectious TB patients; (iii) clinical algorithms for diagnosing smear-negative TB disease in patients seeking care (“rule-in algorithms”); (iv) screening algorithms for excluding TB disease in HIV-infected individuals eligible for preventive therapy (“rule-out algorithms”); and (v) programmatic provision of second-line treatment for multidrug-resistant TB. All were recommended over the past decade [13] [14] [15] [16] [17]; several have been updated since. For each of these interventions, we appraised published studies critically with respect to their objectives, the designs used (how were these questions addressed?), the settings in which they were performed, and their generalizability, giving particular attention to the extent to which the study findings reflected the conditions of, and the patient populations covered by, routine TB services.

## Methods

For each of the five interventions we searched the MEDLINE, EMBASE, Web of Science, and several regional databases (Index Medicus for the Eastern Mediterranean Region, SaudMed, INDMED, HERDIN, Thai Index Medicus, LILACS, African Index Medicus, Koereamed Medicus, Aidsthaidata) for research papers published between 1 January 1990 and 31 March 2012 following a predefined protocol (Texts S1 and S2). All databases searched are available online; we only used databases from researchers that had been peer reviewed and published, and we only included published studies. To maximize the number of publications evaluating effectiveness, delivery, and cost-effectiveness of these interventions, we initially included all publications identified by key word searches (Text S3). For each intervention, two reviewers (EO and FC) independently selected publications from this list on the basis of titles and abstracts, applying preset criteria (Box 1). Additional manual search of the reference lists of reviews was performed; publications thus identified were checked with the initial selections and added if lacking. Since the International Journal of Tuberculosis and Lung Disease has published extensively on the subject of interest, we performed an additional manual search of this journal on a randomly selected 10% of its issues to check if the database search included all relevant titles; we found no publications that were not identified in our initial searches. Only papers written in English, French, Spanish, Portuguese, or German were included.

**Box 1. Selection Criteria for Papers**

Inclusion criteria

- full-text papers reporting on human studies performed in low- or middle-income countries
- abstract in English; publication written in English, French, Spanish, German, or Portuguese

General exclusion criteria

- case reports
- publications that did not address the selected interventions and/or evaluations of these interventions
- mathematical or decision modeling studies not directly based on observations on the intervention concerned (hypothetical cohort models)
- costing studies without an effectiveness component
- reviews

Intervention-specific exclusion criteria

1. *IPT among HIV-infected individuals:*
  - use in immunocompromised individuals other than HIV-infected, including silicosis in low HIV prevalence populations
2. *IPT among household contacts:*
  - use in immunocompromised individuals other than HIV-infected
3. *Clinical algorithms for diagnosing smear-negative TB:*
  - use in immunocompromised individuals other than HIV-infected
  - clinical studies limited to specific diagnostic tools (e.g., bronchoscopy, PCR) and not addressing combinations of tools (i.e., diagnostic algorithms)
  - proof-of-principle studies of diagnostic methods
  - studies reporting the sensitivity of diagnostic tools only
  - studies only assessing predictors of smear-negative TB (i.e., not evaluating or developing a clinical algorithm)
4. *Screening algorithms for excluding smear-negative TB:*
  - as for 3
  - TB prevalence studies/surveys not evaluating diagnostic methods or algorithms or doing so without a reference standard
5. *Second-line treatment of MDR-TB:*
  - pharmacological studies
  - studies specifically aimed at assessing effects of drug resistance on treatment outcomes
  - studies on resistance prevalence and patterns; studies on diagnosis of drug-resistant TB
  - genetic studies
  - retrospective case series reporting outcome data on fewer than 50 patients
  - studies on surgical interventions only describing the patients who had surgery (studies that describe a cohort of MDR patients of whom a portion had surgery were included)

Data from all selected papers were entered into a MS Excel database (Microsoft Corp), including study objectives, design, settings, and results. Two reviewers (SvK and FC) independently appraised each included publication; disagreement was solved by consensus (FC). In addition to the key evaluation criteria (objective[s], design, setting, generalizability), for studies that reported health outcomes we appraised the extent to which they addressed effectiveness rather than efficacy (Box 2). Our review did not aim to summarize the results of the studies, and by its nature included studies of highly varying designs and methodologies. Therefore we did not perform any quality assessments.

**Box 2. Categorization Criteria of Reviewed Studies**

1. Study objective(s)

Studies were categorized as *evaluating effects of the intervention on health outcomes*; its *delivery*; its *cost-effectiveness*; or *other*. Further categorization was specific for the intervention under review. Only those objectives were categorized that were specifically mentioned in the paper; more than one objective was allowed.

2. Study design

Studies were categorized as *comparative and non-comparative studies*. Comparative studies were defined as studies that compared outcomes for different interventions, with or without experimental design and randomized allocation. Non-comparative studies were defined as cohort studies or cross-sectional studies that did not compare interventions. Papers reporting on non-comparative analyses within comparative studies were recorded as non-comparative.

3. Study setting

Studies were categorized according to the country where the study was conducted (grouped into global regions), to mid-period (2005) estimated incidence of TB, to mid-period prevalence of HIV infection among TB patients [62], and/or to prevalence over the period 2000–2009 of multidrug resistance among TB patients [63].

In addition, the study location was categorized as a *research setting*, a *mixed routine/research setting*, or a *routine setting*. We defined a research setting as one with extensive clinical and laboratory research facilities with strong potential for research-driven diagnostic, treatment, and follow-up procedures; a routine setting as one with routine clinical and laboratory facilities and procedures only; and a mixed setting as a combination of the two, e.g., a routine treatment setting with research-driven follow-up procedures. For studies of diagnostic algorithms we in addition categorized the studies by the patient populations included.

4. Generalizability of the study

Study results were considered *generalizable irrespective of epidemiological or health care setting* if they were likely not affected by setting-specific factors, *generalizable to similar epidemiological or health care settings* if they were likely affected by factors that are common across settings (e.g., HIV infection prevalence), and *not generalizable beyond the country or setting in which the study was done* if such factors were highly setting-specific (e.g., non-completion due to migration).

5. Efficacy versus effectiveness

Studies that reported health outcomes were categorized as *primarily assessing efficacy*, *primarily assessing effectiveness*, or *mixed*. Efficacy studies were defined as studies with strict protocol-defined in- and exclusion criteria of study subjects and optimized adherence or diagnostic procedures. Effectiveness studies were defined as studies that applied routine or programmatic criteria for in- and exclusion criteria of study subjects and routine measures for enhancing adherence [64].

Finally, we evaluated the distribution of the studies according to their geographical location in four global regions (Central/South America, sub-Saharan Africa, Middle East and South Asia, and East and Southeast Asia), their type (effectiveness, cost-effectiveness and delivery studies), their design (comparative or non-comparative), and the setting in which they were conducted (routine/programmatic, research, or mixed routine-research). This assessment allowed us to assess the general landscape of interventions arising from these data, thereafter referred to as “the research landscape.” Because of the high MDR-TB prevalence and specific organization of the TB control system, for second-line treatment we added the former Soviet Union as a separate region.

The funders of this study (the Stop TB Partnership and Global Fund to fight AIDS, Tuberculosis and Malaria) were involved in study design and preparation of the manuscript but did not influence the data collection, analysis, or decision to publish.

## Results

### Isoniazid Preventive Therapy

**Figure 1:**
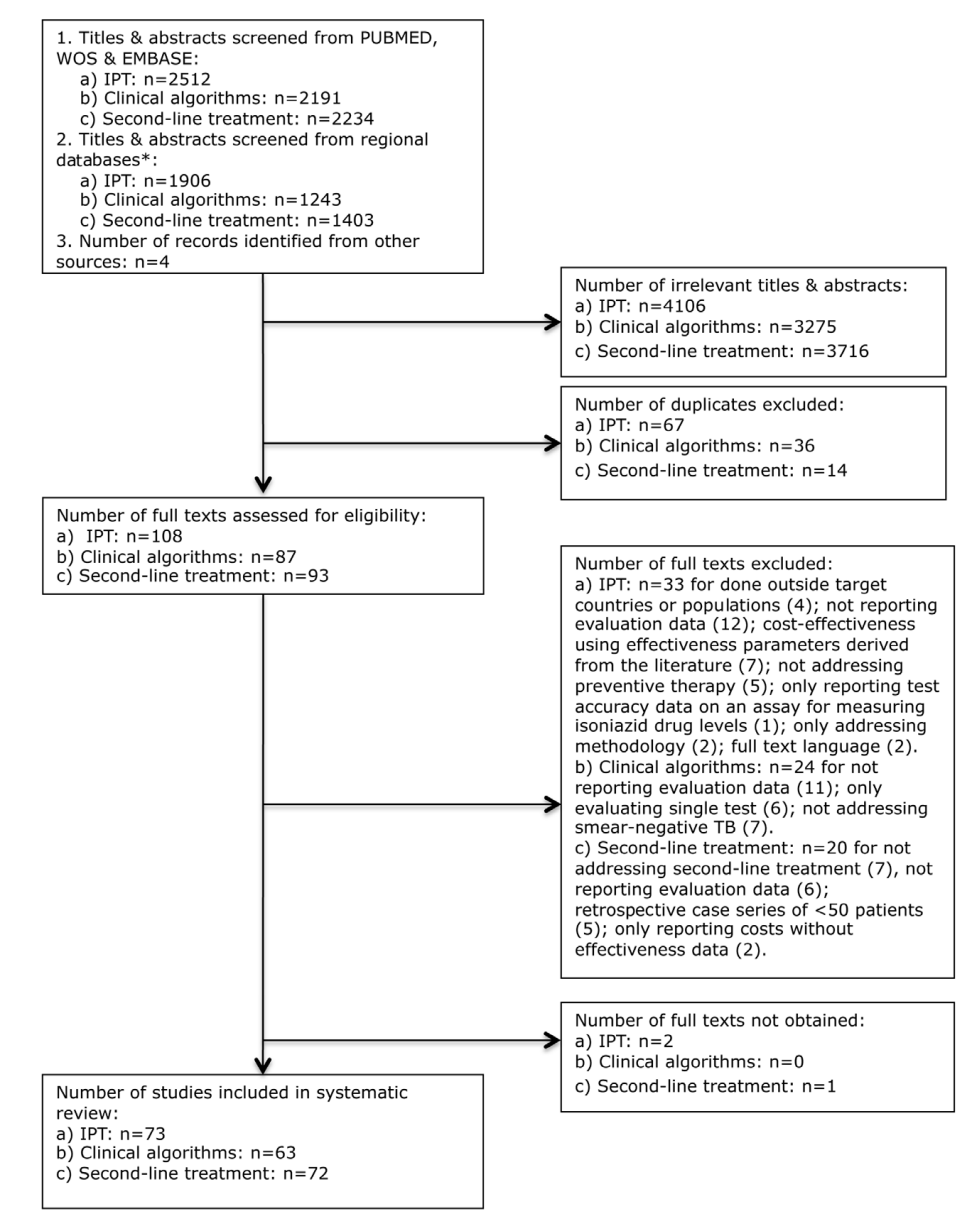
Flow chart for selection of articles for isoniazid preventive therapy (a), clinical algorithms for diagnosis/screening of smear-negative pulmonary TB (b), and second-line TB treatment (c).

Of 4,418 titles and abstracts screened we included 73 studies in the analysis (Figure 1), of which two were identified from regional databases only. Fifty-seven studies addressed IPT in HIV-infected individuals, 14 addressed IPT in household contacts (13 in children, one in all age groups), and two addressed IPT in miners in South Africa. Since HIV prevalence in these study populations was high we included the latter study in the HIV category, bringing the total number of HIV studies to 59. Forty-seven of the 73 studies considered the association of IPT with health outcomes, 44 in HIV infected individuals, and three in household contacts. Of the 44 studies involving HIV-infected individuals that addressed effects on health outcomes, 16 were considered efficacy studies and 12 effectiveness studies; 16 had elements of both.

Thirty-six of the 73 studies (49.3%; 33 among HIV-infected individuals) assessed the association of IPT with TB incidence or progression of the HIV infection, with 34 (also) addressing adverse effects of IPT. Six studies reported drug resistance patterns among TB cases occurring during or after IPT, all among HIV-infected individuals, including four individually randomized trials, and one comparative and one non-comparative cohort study. Forty-eight (65.8%) studies investigated aspects of care delivery including seven in which this was done as part of an individually randomized trial, and four (6.6%; three for HIV-infected individuals, and one for household contacts) that assessed the effects of interventions to improve completion of, or adherence to, IPT. Cost-effectiveness was examined in four studies (6.6%); one additional study provided costing data (Table 1).

**Table 1:** Results for studies on preventive therapy in HIV-infected individuals and in household contacts.

Appraisal	HIV Infection, *n* = 59		Household Contacts, *n* = 14		Total, *n* = 73	
	Major Category	Minor Category	Major Category	Minor Category	Major Category	Minor Category
**Year of publication**						
1990–1995	2		0		2	
1996–2000	11		1		12	
2001–2005	11		2		13	
2006–2012	35		11		46	
**Objective**						
Effects on health outcomes	44		3		47	
Evaluation of IPT for preventing TB or progression to AIDS	33		3		36	
Comparing IPT versus no IPT only		11		0		11
Comparing various regimens and dosing schedules		9		0		9
Comparing various durations		2		0		2
Comparing various patient groups^a^		1		0		1
Comparing IPT versus HAART with or without IPT		4		0		4
No comparison		6		3		9
Frequency of and risk factors for adverse effects^b^	31		3		34	
Drug resistance among TB cases during or after IPT^b^	6		0		6	
Delivery	35		13		48	
Evaluation of IPT completion/adherence rate^c^						
Comparing different regimens		2		1		3
Comparing interventions for enhancing completion and/or adherence		1		0		1
Frequency of and risk factors for non-completion or non-adherence^d^		28		6		34
Comparison of various IPT enrolment methods		1		1		2
Barriers to implementation		1		0		1
Assessment of practices		2		5		7
Cost-effectiveness	4		0		4	
**Study design**						
Comparative studies	31		2		33	
Individually randomized trials		19		0		19
Group-randomized trials		0		0		0
Non-randomized cohort comparisons		10		1		11
Before–after comparisons		1		0		1
Other		1		1		2
Non-comparative studies	28		12		40	
Prospective cohort studies		19		4		23
Retrospective cohort studies		5		5		10
Cross-sectional clinical studies		2		1		3
Surveys		2		2		4
**Setting**						
Estimated TB incidence per 100,000 population (2005)	57^e^		14		71^e^	
<50		2		1		3
50–99		5		2		7
100–299		7		3		10
≥300		43		8		51
Estimated HIV prevalence among TB patients (2005)	57^e^		14		71^e^	
<5%		3		4		7
≥5%		54		10		64
Study location	59		14		73	
Research setting		22		0		22
Mixed research – routine setting		10		2		12
Routine setting		27		12		39
**Generalizability**						
Study results are generalizable:	59		14		73	
Irrespective of epidemiological or health care setting		31		4		35
To similar epidemiological or health care settings		18		7		25
Not beyond national/local setting		2		2		4
Other						
Small sample size		3		0		3
Assessment of operational issues in research setting		5		1		6
**Effectiveness versus efficacy**						
Methods aimed at establishing (relevant studies)	44^f^		3^f^		47^f^	
Efficacy		16		0		16
Effectiveness		12		3		15
Mixed		16		0		16

a For example, comparing patients with positive versus negative tuberculin skin tests.

b Including studies that addressed effects on treatment outcomes.

c Studies testing a hypothesis about measures to improve treatment completion or adherence.

d As specific study objective, no hypothesis testing about measures to improve treatment completion or adherence.

e Two multi-country studies situated at locations with different TB incidences and HIV prevalence; one of these in various regions.

f Number of studies evaluating effects on health outcomes.

HAART, highly active antiretroviral treatment.

**Figure 2:**
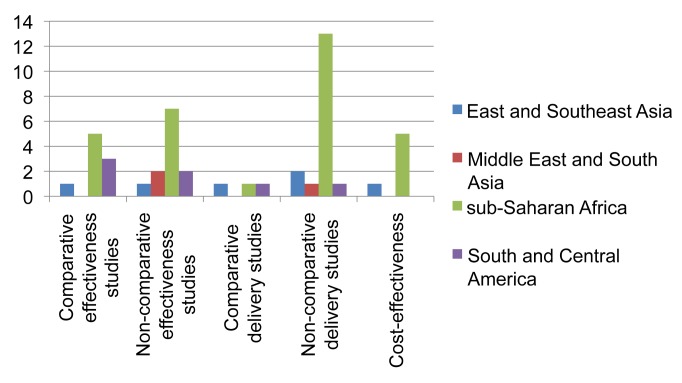
Distribution of published studies on isoniazid preventive therapy of HIV-infected individuals, by geography, objective, and study setting. Effectiveness studies relate to studies designed to address effectiveness as well as mixed effectiveness-efficacy for health-related outcomes, done in routine or mixed routine-research settings. Delivery studies relate to studies designed to address treatment completion and adherence, practices, and organization of services. Two comparative and two non-comparative delivery studies were also included as effectiveness studies, and two cost-effectiveness studies were also included as delivery studies.

**Figure 3:**
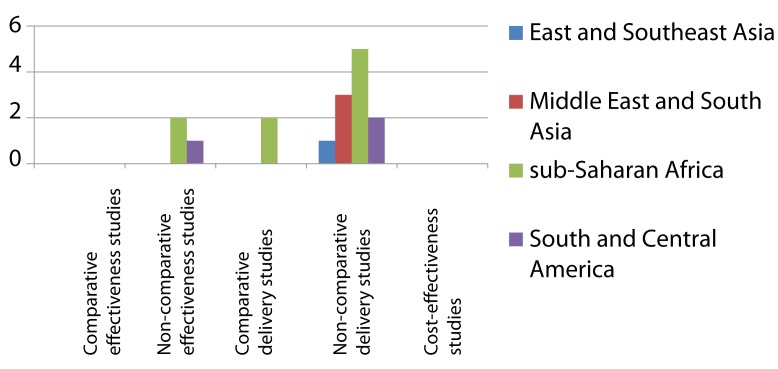
Distribution of published studies on preventive therapy of household contacts of infectious TB patients, by geography, objective, and study setting. Effectiveness studies relate to studies designed to address effectiveness as well as mixed effectiveness-efficacy for health-related outcomes, done in routine or mixed routine-research settings. Delivery studies relate to studies designed to address treatment completion and adherence, practices, and organization of services. Two non-comparative delivery studies were also included as effectiveness studies.

Thirty-three studies followed a comparative research design (45.2%), including 19 individually randomized trials (all among HIV-infected individuals). Although two studies included data from group-randomized trials [18],[19], none reported analyses of trial outcomes. Twenty-three prospective and ten retrospective cohort studies used a non-comparative design. Sixty-four of the 71 single-country studies (90.1%) were conducted in countries with HIV prevalence among TB patients of ≥5%. The majority of these studies were undertaken in sub-Saharan Africa (45; 61.6%) and the Americas (14; 19.2%) (Figures 2 and 3). Two-thirds of all studies (50, 68.5%) were conducted in just five countries, namely South Africa (22), Uganda (9), Brazil (8), Thailand (6), and Haiti (5), and the majority by the same research groups. Twenty-two (30.1%) studies were in research settings, and 39 (53.4%) in routine settings.

We categorized the results of 35 studies (47.9%) as generalizable irrespective of setting, 25 (34.2%) as generalizable to similar epidemiological and health care settings, and four as not generalizable beyond local or national setting.

There was a sharp increase in numbers of studies from 2006 onwards, when 46 of the 73 studies (63.0%) were published. Eleven effectiveness studies and 14 studies addressing IPT delivery were published in 2010 and 2011 only.

The research landscape shows that the majority of effectiveness studies on IPT among HIV-infected individuals were undertaken in sub-Saharan Africa (Figure 2). Comparative effectiveness studies done outside research settings for IPT in HIV-infection (*n* = 9) were mainly limited to South America and sub-Saharan Africa. For IPT in contacts (Figure 3) there is limited data on effectiveness, none of these arising from comparative studies. Comparative intervention studies on delivery aspects and cost-effectiveness studies are rare for both interventions. There is little published evidence for scale-up of IPT from outside Africa or the Americas.

### Clinical Algorithms for Detecting Pulmonary Tuberculosis

Of 3,434 titles and abstracts screened we included in the analysis 63 studies examining clinical algorithms (Figure 1); two were identified from regional databases only. Forty-four studies primarily addressed rule-in algorithms for smear-negative TB, and 19 primarily addressed rule-out algorithms for any pulmonary TB. Of the 63 included studies, 19 (30.2%) evaluated predefined diagnostic algorithms: 15 for rule-in and four for rule-out. Twenty-five studies assessed clinical predictors (39.7%; 11 for rule-in, 14 for rule-out). Of these, eight rule-in studies and 12 rule-out studies used the resulting predictions to develop a clinical scoring system, a clinical algorithm, or a screening algorithm. For seven rule-out studies the scoring system or algorithm was primarily based on symptoms. In addition, one of the rule-in studies evaluated the developed clinical algorithm on a separate partition of the dataset [20]. Eleven (17.5%) studies evaluated diagnostic procedures added to clinical algorithms, of which 3 also assessed cost-effectiveness. Ten studies (15.9%, all rule-in) addressed delivery issues.

The majority of the studies had non-comparative prospective cohort (28, 44.4%) or cross-sectional (26, 41.3%) designs. Two studies compared predefined rule-in algorithms against standard practice, both in a before–after design: one compared the proportions of smear-positive diagnoses before and after introducing a locally developed clinical algorithm in Ethiopia using routine notification data [21], and the other study compared hospitalization and mortality among severely ill HIV-infected individuals suspected to have TB, before and after introducing the WHO algorithm for diagnosis of smear-negative TB [22] in routine practice in South Africa [23]. Four other recent studies evaluated the 2007 WHO algorithm for ruling in smear-negative TB; one compared it to the 2003 WHO algorithm in Uganda [24], and the remaining three (from Brazil, Cambodia, and South Africa) used a non-comparative cross-sectional or retrospective design [25]–[27]. One study evaluated the most recent WHO-recommended algorithm for ruling out TB in HIV-infected individuals in Vietnam using a non-comparative design [28]. Of the rule-in studies, 34 (77.3%) included TB suspects, while eight (20.5%) only included smear-negative TB patients, which did not allow assessment of the specificity of the algorithm (Table 2).

**Table 2:** Results for studies on clinical algorithms for diagnosis of smear-negative TB in patients presenting with symptoms (“rule-in”) and for screening of HIV-infected individuals (“rule-out”).

Appraisal	Rule in, *n* = 44		Rule out, *n* = 19		Total, *n* = 63	
	Major Category	Minor Category	Major Category	Minor Category	Major Category	Minor Category
**Year of publication**						
1990–1995	2		0		2	
1996–2000	7		0		7	
2001–2005	12		3		15	
2006–2012	23		16		39	
**Objective**						
Effects on health outcomes	34		19		53	
Evaluation of predefined algorithms		15^a^		4		19
Evaluation of diagnostic procedures						
With developing one or more algorithms		8		12		20
Without developing one or more algorithms		3		2		5
Evaluation of diagnostic procedures added to clinical algorithms^b^		8		1		9
Delivery	10		0		10	
Improvement of sputum collection		6		0		6
Improvement of smear examination		2		0		2
Assessment of practices		2		0		2
Cost-effectiveness	2^C^		1^c^		3^c^	
**Study design**						
Comparisons of algorithms	4		0		4	
Non-comparative studies	40		19		59	
Prospective cohort studies		19		9		28
Retrospective cohort studies		3		1		4
Cross-sectional studies		17		9		26
Surveys		1		0		1
Study population	44		19		63	
All TB suspects		14		6		20
All smear-negative TB suspects		20		3		23
Smear-negative TB patients		8		0		8
All HIV+ patients		0		4		4
All patients eligible for IPT cf. programmatic criteria		0		3		3
Patients notified with pneumonia (surveillance)		1		0		1
Patients notified with TB (surveillance)		1		0		1
Community-based survey		0		2		2
Prison survey		0		1		1
**Setting**						
Estimated TB incidence per 100,000 population (2005)	44		18^d^		62^d^	
<50		0		0		0
50–99		6		1		7
100–299		12		3		15
300+		26		14		40
Estimated HIV prevalence among TB patients (2005)	44		17^e^		61^e^	
<5%		12		1		13
5% or more		32		16		48
Study location	44		19		63	
Research setting		2		0		2
Mixed research – routine setting		10		3		13
Routine setting: 1–3 clinics		25		9		34
Routine setting: >3 clinics		7		5		12
Population based		0		2		2
**Generalizability**						
Study results are generalizable:	44		19		63	
Irrespective of epidemiological or health care setting		13		13		26
To similar epidemiological or health care settings		25		6		31
Not beyond national/local setting		5		0		5
Other:						
Small sample size, unclear patient selection		1		0		1
**Effectiveness versus efficacy**						
Methods aimed at establishing:	34^f^		19^f^		53^f^	
Efficacy		2		1		3
Effectiveness		15		9		24
Mixed		17		9		26

a Excluding one that generated an algorithm that was subsequently evaluated in a separate (prediction) dataset.

b Additional diagnostic procedures evaluated include bronchoalveolar lavage, nasopharyngeal aspirate, stool culture, fluorescence microscopy, PCR, urinary lipoarabinomannan, microscopic observation of drug susceptibility (MODS), endobroncheal ultrasound, repeat of smear examination after 1 mo.

c These studies also assessed effects on health outcomes.

d Excluding one multi-country study [65].

e Excluding two multi-country studies [65],[66].

f Number of studies evaluating effects on health outcomes.

**Figure 4:**
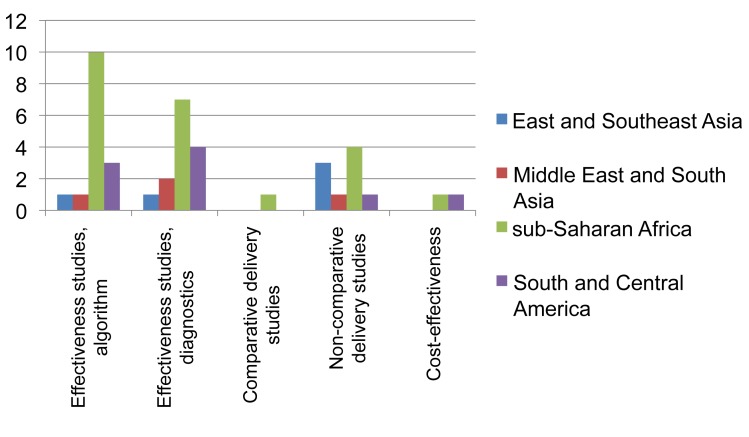
Distribution of published studies on clinical algorithms for diagnosing smear-negative TB in patients presenting with symptoms (“rule-in”), by geography, objective, and study setting. Effectiveness studies, algorithm relate to studies designed to evaluate predefined clinical algorithms, and effectiveness studies, diagnostics to studies designed to evaluate combined diagnostic methods, both for diagnosing smear-negative TB among TB suspects done in routine or mixed routine-research settings. Delivery relates to studies designed to address diagnostic practices and improvement of smear examination or sputum collection to improve diagnosis of smear-negative TB. One study evaluated both combined diagnostic methods and a predefined clinical algorithm. Two cost-effectiveness studies were also included as evaluations of combined diagnostic methods.

**Figure 5:**
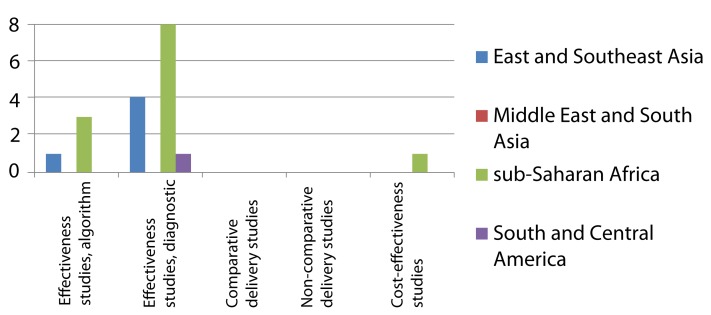
Distribution of published studies on clinical algorithms for screening for smear-negative TB in HIV-infected individuals (“rule-out”), by geography, objective, and study setting. Effectiveness studies, algorithm related to studies designed to evaluate predefined clinical algorithms, and effectiveness studies, diagnostics to studies designed to evaluate combined diagnostic methods, both for excluding TB among HIV-infected individuals and done in routine or mixed routine-research settings. One cost-effectiveness study was also included as evaluation of combined diagnostic methods.

Forty-eight (78.7%) of the 61 single/similar-country studies were performed in countries with HIV prevalence among TB patients of ≥5%. Again, the majority of these studies were conducted in sub-Saharan Africa (40; 65.6%) (Figures 4 and 5). Forty-six (73.0%) studies took place in routine settings and 15 in research or mixed research-routine settings. We categorized the results of 26 studies (41.3%) as generalizable irrespective of setting, 31 (49.2%) as generalizable to similar epidemiological and health care settings, and five as non-generalizable beyond local setting (Table 2). Out of the 53 studies with health outcomes, 24 were categorized as primarily assessing effectiveness (45.3%), 26 (49.1%) as having elements of both effectiveness and efficacy, and three as evaluating efficacy only.

The number of studies increased from 2006 onwards, when 39 of the 63 studies (61.9%) were published. Of 21 studies that evaluated predefined algorithms, eight were published in 2010 or 2011.

The landscape for rule-in diagnosis (Figure 4) shows that while effectiveness studies of diagnostic algorithms or combined diagnostics done in the relevant study population, i.e., individuals suspected to have TB, have been published from sub-Saharan Africa (*n* = 15), very few have come from other geographical regions. Only four of such effectiveness studies had a comparative design. There have been few studies on delivery aspects or cost-effectiveness, and again, there are very limited data from the Middle East/South Asian region. For rule-out diagnosis (Figure 5), there have been a number of studies on specific combined diagnostics including symptom screening, but few that evaluated existing algorithms; these studies were almost exclusively from sub-Saharan Africa and East/Southeast Asia. No studies were published on delivery aspects and only one reported cost-effectiveness data.

### Programmatic Provision of Second-Line Treatment for Multidrug-Resistant Tuberculosis

Of 3,637 titles and abstracts screened, we included 72 articles in the analysis (Figure 1), of which three were identified from regional databases only. The large majority (59, 81.9%) were non-comparative retrospective or prospective cohort studies that evaluated outcomes for individualized (i.e., guided by the individual drug resistance pattern) or standardized (i.e., guided by resistance patterns in the population) treatment (Table 3).

**Table 3:** Results for studies on provision of second-line treatment for multidrug-resistant TB.

Appraisal	Total, *n* = 72	
	Major Category	Minor Category
**Year of publication**		
1990–1995	0	
1996–2000	4	
2001–2005	15	
2006–2011	53	
**Objective**		
Effects on health outcomes		
Evaluation of treatment outcomes of second-line treatment	59	
Individualized regimen		42
Standardized regimen		14
Individualized and standardized regimens^a^		2
Not reported		1
Frequency of and risk factors for adverse effects	17^b^	
Drug resistance amplification, re-infection	1	
Delivery	13^c^	
Evaluation of treatment completion and/or adherence^d^		
Comparing interventions for enhancing completion/adherence		5^c^
Frequency of and risk factors for non-completion or non-adherence^e^		3
Evaluation of role of nurses in treatment support		1
Description drug ordering system		1
Implementation and coverage of second-line treatment		1
Analysis of treatment enrolment		1
Comparison of methods for treatment monitoring		1
Cost-effectiveness	2^f^	
**Study design**		
Design		
Comparative studies	7	
Individually randomized trials		2
Group-randomized trials		0
Non-randomized cohort comparisons		3
Before–after comparisons		2
Non-comparative studies	65	
Prospective cohort studies		22
Retrospective cohort studies		40
Case-control studies		1
Other		2
**Setting**		
Estimated TB incidence per 100,000 population (2005) [62]	72	
<50		8
50–99		18
100–299		36
300+		8
Various		2
Estimated MDR prevalence among new TB patients (1994–2007) [63]	72	
<3%		41
3–6%		18
>6%		11
various		2
Study location	72	
Research setting		0
DOTS-Plus pilot project		21
Routine—specialized clinic		27
Routine – programmatic		21
Various		3
**Generalizability**		
Study results are generalizable:	72	
Irrespective of epidemiological or health care setting		53
To similar epidemiological or health care settings		19
Not beyond national/local setting		0
Other		0
Methods aimed at establishing (relevant studies):	62^g^	
Efficacy		4
Effectiveness		57
Mixed		1

a Studies covering multiple sites or periods.

b Including eight studies that evaluated treatment outcomes.

c Including two studies that did so by (also) evaluating treatment outcomes.

d Studies testing a hypothesis about measures to improve treatment completion or adherence.

e As specific study objective, no hypothesis testing about measures to improve treatment completion or adherence.

f Also addressing treatment outcomes.

g Number of studies evaluating effects on health.

These articles included 11 studies that addressed XDR-TB, either uniquely or in combination with non-XDR MDR-TB, and nine that were done in a patient population with an HIV infection prevalence of ≥5%. Only one cohort study compared different second-line drug regimens for treatment outcomes using a non-randomized, group-wise before–after design [29]. Another cohort study compared outcomes for centralized versus decentralized treatment [30]. Two articles reported different analyses on the same patient cohort [31],[32].

Fourteen studies (19.4%) addressed delivery issues, including five that assessed the effects of specific interventions for improving treatment adherence in a comparative design: two randomized-controlled trials comparing the effects of clinical pharmacist-directed patient education [33] and of telephone-assisted support [34]; a before–after comparison of decentralized patient management [35]; another before–after comparison of community- versus hospital-based treatment [36]; and a semi-quantitative case study of psychosocial support groups [37].

All of these studies also assessed predictors of successful or poor treatment outcomes, and two included cost-effectiveness analyses in programmatic settings. Eight of the cohort studies of treatment outcomes and nine additional studies specifically assessed frequency of and risk factors for adverse effects. One cohort study also reported on amplification of drug resistance and *M. tuberculosis* re-infection during treatment [38].

Of the 62 selected studies of second-line treatment that evaluated the associated health outcomes, almost all (58, 93.5%) were categorized as assessing effectiveness rather than efficacy.

Thirty-nine studies (54.2%) were from just five countries: Peru (11), South Africa (nine), South Korea (eight), India (six), and Latvia (five). This set of studies reflected to a large extent the research groups involved: 27 of these studies involved three research groups. Twenty-one (29.2%) studies were done in pilot projects of the “DOTS-Plus” approach to second-line treatment, and 48 (66.7%) in routine settings, including specialized clinics for 27 studies and programmatic settings for 21.

We categorized 53 studies (73.6%) as generalizable irrespective of setting. These were mainly cohort studies of treatment outcomes that were primarily determined by drug resistance pattern and drug regimen, except four that had highly setting-specific elements with regard to treatment completion (e.g., involving prison populations). All remaining studies were categorized as generalizable to similar epidemiological and health care settings.

All included studies were published after 1995; about three-quarters (53, 73.6%) were published from 2006 onwards. Of the 35 effectiveness studies done in programmatic of pilot settings, 13 were published since 2010.

**Figure 6:**
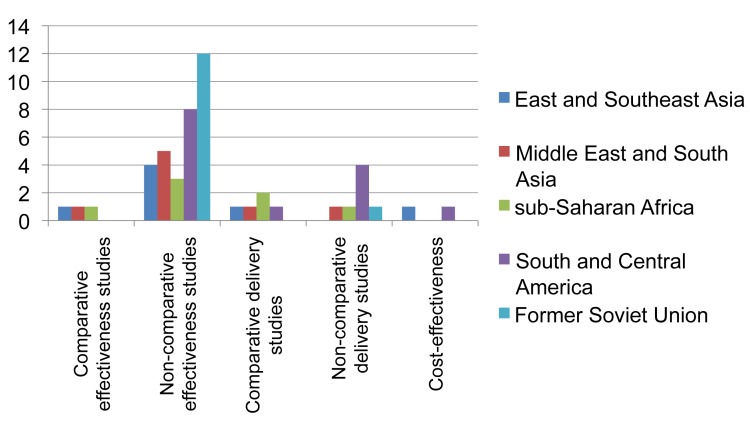
Distribution of published studies on provision of second-line treatment for drug-resistant TB, by geography, objective, and study setting. Effectiveness studies relate to studies designed to address effectiveness as well as mixed effectiveness-efficacy for health-related outcomes, done in programmatic settings or “DOTS-Plus” pilots. Delivery relates to studies designed to address treatment completion and adherence, and organization of services. One non-comparative delivery study and two cost-effectiveness studies were also included as effectiveness studies.

The landscape arising from these data (Figure 6) shows that while non-comparative effectiveness studies (case series) in programmatic or pilot settings have been published from all regions of the world, studies comparing various interventions for their effectiveness outside research settings have been rare (*n* = 3). Studies on care delivery aspects are infrequent and those published are mainly from Peru. Studies specifically assessing cost-effectiveness are rare.

## Discussion

Our systematic review demonstrates the paucity of published evidence for scale-up of five selected interventions for TB control in real-life conditions in various epidemiological and health care settings. In addition, the few published studies had limitations with regard to their design, their geographical distribution, and the settings in which they were conducted: studies aimed at assessing effectiveness rather than efficacy mainly had non-comparative designs, were geographically clustered (primarily in sub-Saharan Africa), and were often not done in sites or patient populations that reflect the routine health care settings in which the interventions need to be applied. Of the 208 reviewed studies for all five interventions combined, only about one-fourth (54 studies) evaluated ways of delivering these interventions in routine health care settings, and only nine assessed their cost-effectiveness, showing the limited evidence accrued for guiding their programmatic scale-up.

While these shortcomings are specific to each of these five interventions, there are a number of shared features. True real-life studies of effectiveness in programmatic settings are rare. While several studies assessed the efficacy of IPT under optimal conditions (such as in research settings) or in selected groups of patients, very few studies evaluated the effectiveness of IPT under routine conditions. Likewise, although in recent years a number of studies evaluating clinical algorithms for rule-in diagnosis of smear-negative TB or for rule-out of TB among HIV-infected individuals in programmatic settings have been published, the geographical distribution of these studies is patchy. For example, South Asia is consistently underrepresented.

In addition, very few studies have evaluated methods to optimize the delivery of the intervention. For example, while observational cohort studies of delivery of second-line treatment have been important to show its feasibility in resource-poor settings and identify best practices of treatment adherence, there are few published direct comparisons of such practices, and very few have used a comparative design.

Another common feature is the paucity of published economic evaluations of the recommended interventions according to our selection criteria (nine published cost-effectiveness analyses only, including none on IPT for contacts and only one on rule-out algorithms). This is not to say that no other cost-effectiveness analyses have been published. We found seven additional papers on cost-effectiveness modelling (all on IPT in HIV-infected individuals), but these studies modelled hypothetical cohorts that were not or only partially based on effectiveness and costing data as observed within single studies. Since these models tend to reflect ideal rather than real-life conditions, we considered these less relevant for decisions about scale-up at the country level.

Finally, and most importantly, relatively few studies had appropriate methods to evaluate interventions or the models to deliver these interventions. While a number of study designs can be used to demonstrate effectiveness of interventions, experimental or quasi-experimental methods with a comparative element are generally considered to provide the strongest evidence, particularly when the intervention is compared to existing practice. Since health interventions are often applied at the group level (e.g., entire clinics), such comparative studies preferably have randomized group-wise allocation [39]. This study design, also known as group- or cluster-randomized trial, has various extensions that allows study of intervention effects during implementation (e.g., the stepped wedge design) [40]–[42]. Although we became aware of two group-randomized comparative studies on IPT in HIV infection that are underway in Brazil and South Africa, respectively [18],[19], we found no reports of group-randomized trials for any of the five interventions over the last decade. Increasingly used in other disease areas [43], this study design has found little application in TB. We believe this is a missed opportunity as the standardized diagnosis, treatment, and recording of the classical DOTS programs are particularly well suited for such studies, e.g., by randomizing diagnostic and treatment centres to one or the other intervention model [7],[44]. In addition, when applied across programs, such standardization allows multi-country studies of similar approaches in different settings—such as the multi-site study on provision of second-line treatment by Nathanson et al. [45],[46].

It should be noted, however, that (quasi-)experimental designs have potential drawbacks with regard to the representativeness of the study results for routine health care setting, as the research investment required tends to alter health care practice. Such interference may nonetheless be limited by issues such as basing data collection to a large extent on routine recording and reporting, and not collecting any clinical material beyond the intervention under study, which may also obviate the need for individual informed consent.

We found only a small number of studies that addressed delivery issues such as adherence to treatment or improved operations of existing diagnostics. This may be because these studies are conducted and reported locally, but not published in the peer-reviewed journals covered by our search, and because of publication bias leading to preferential publication of studies reporting successful outcomes [47].

Our review has a number of strengths. It assessed several TB control interventions in a single framework, allowing us to separate the characteristics that are common in the search for evidence for scale-up from those that are specific for each intervention. The framework that we applied categorizes studies according to a set of verifiable evaluation criteria, and even though the appraisal of study setting and generalizibility of the study results had subjective elements, we defined these in a way that allows reproducibility for similar exercises on different interventions or diseases.

Our approach also has a number of limitations, in addition to those already mentioned. The interventions we selected do not cover all programmatic interventions in TB. However, the interventions we selected have all been recommended by the WHO and received attention as being poorly implemented, [48]–[51] despite being evidence-based with data showing that, under controlled circumstances, they improve prevention, diagnosis, or treatment of TB. In addition, our review only covered the period from 1990 to early 2012, and we may have missed studies published earlier. This time range could explain the small number of studies identified on IPT for household contacts, as this intervention was already recommended by WHO before 1990, based on randomized controlled trials of 12 or more months of isoniazid treatment among household contacts of infectious TB patients conducted in the 1960s in the USA, Puerto Rico, Mexico, Kenya, and The Philippines [52]–[55], as well as a community study in Alaska [56]. However, this timeframe was before WHO's DOTS Strategy was launched mid-1990s [57], and most studies on effectiveness, delivery or cost-effectiveness of chemoprophylaxis of household contacts in DOTS-style TB control programs should have been published after that. The other interventions were recommended within the last decade, and impact studies are likely to have been done and published in the study period. Moreover, we found, for all interventions combined, only four studies published in the period 1990–1995, making it unlikely that our restriction of the review period caused us to miss studies that would have altered our conclusions.

Finally, although our search in addition to three global databases covered the most important literature databases for India, Africa, South- and Central America, the Arab subcontinent, the Philippines, Thailand, and South Korea, we did not search for publications in major languages such as Chinese, Arab, and Russian. However, among the over 4,000 titles we screened in regional databases, we found only five publications that had not yet been identified from the global databases, indicating that a more extensive search is unlikely to yield many more relevant publications and fundamentally different conclusions.

A detailed operational research agenda to address the implementation of WHO policies for TB control at country level was recently issued [58],[59]. This review shows a number of gaps in the realization of this agenda. More studies are needed to show the effectiveness of IPT, including its effect on development of drug resistance, in HIV-infected persons as a programmatic intervention in countries representing a broad range of epidemiological and health system settings, notably in Asia. More studies are needed on IPT of household members outside of the context of HIV infection, and should include evaluation of cost-effectiveness. In addition, studies should assess approaches that enhance access to and adherence with each intervention as important delivery aspects. For clinical diagnosis of smear-negative TB, there is a need for studies that evaluate and compare effectiveness, delivery, and cost-effectiveness of rule-in and rule-out algorithms, especially outside sub-Saharan Africa. Furthermore, for provision of second-line treatment, different delivery models aimed at enhancing treatment adherence and management of adverse effects need to be evaluated in varied settings and compared for programme scalability.

This review showed the paucity of published data on the effectiveness, delivery, and cost-effectiveness of a selected number of new interventions in TB control in contexts where they need to be implemented. This lack of “evidence for scale-up” may be an important cause of the shortfall in implementation of these interventions in many countries. The recent diagnostic breakthrough brought about by the development of the Xpert MTB/RIF, a fully automated cartridge-based nucleic acid amplification assay that was endorsed by the WHO in December 2010, may catalyse studies on operational aspects of this test, its effectiveness in program conditions, and its cost-effectiveness. Furthermore, this review underlines the need for novel and creative thinking to address the gaps that are occurring between global policy recommendations on new interventions and their real-life implementation in control programs, and that severely hamper efficient TB control. A broad concerted effort is urgently needed to develop operational-research capacity, allocate appropriate resources, and encourage all actors to work together [60] to promote the use of rational and objective-driven operational research in TB control to suitably inform policy making [59] as identified by the Global Plan to Stop TB 2011–2015, which incorporates research as a priority to improve TB control globally [2]. This effort may require funding agencies to reconsider their priorities. The 208 publications that we included in our review constitute only a minute fraction of the 81,854 publications on TB over the review period that were listed in PubMed alone, which included, for example, 591 papers on interferon-gamma release assays that are of very limited use in countries with high TB incidences [61]. Further, it requires not only that more operational studies are conducted, but also that the results are made publicly available, thus placing responsibilities with researchers, funding agencies, and journal editors.

## Supporting Information

Text S1 PRISMA statement.
(DOC)

Text S2 Review protocol.
(DOC)

Text S3 Search strategies, details of included studies, and full reference list.
(DOCX)

## Abbreviations

IPT: isoniazid preventive therapy
TB: tuberculosis
WHO: World Health Organization
