# Supplementary material for: Research on Implementation of Interventions in Tuberculosis Control in Low- and Middle-Income Countries: A Systematic Review
Source: PLoS Med. 2012 Dec 18;9(12):e1001358. doi: 10.1371/journal.pmed.1001358 (PMC3525528; doi:10.1371/journal.pmed.1001358)
Supplement: Text S2 — Review protocol. (DOC) [file pmed.1001358.s002.doc]

**Text S2**

**Implementation research of interventions in tuberculosis control in low and middle income countries: a systematic review.**

**Systematic review protocol**

Date: 30 April 2010

Amended for search period and databases: 1 November 2011.

Investigators

*Frank Cobelens*

*Sanne van Kampen*

*Eleanor Ochodo*

*Rifat Atun*

*Christian Lienhardt*

## Background

There is a need for more and better evidence for decision-making with regard to new interventions in TB control; internationally but also regionally/nationally. Initial evaluations of new interventions under controlled conditions (clinical, laboratory) are important, but also needed are evaluation in real-life, programmatic setting and with respect to patient-important outcomes such as reduced treatment delay, improved cure rate, reduced mortality, quicker regain of ability to work, reduced risk of relapse and drug resistance. Also scalability of the intervention to the level of a TB program is of relevance. This requires real-life data on factors that enable or hamper the delivery of these interventions. Such factors may relate to e.g. measures to improve treatment adherence, optimization of access to interventions, or training requirements of staff. Finally, costs and cost-effectiveness of interventions, when compared to existing control methods or alternative intervention are also important. Together these three areas constitute the “evidence for scale-up” needed as a basis for policy decisions.

Much research in this field is being done but apparently with limited international coordination, and there seems to be substantial diversity in study objectives, designs, settings and relevance. It is thus unclear whether evaluation needs are sufficiently met and optimum use is made of available potential and resources.

Therefore a systematic review will be done of the evaluation landscape to identify current gaps in evidence for scale-up. Since the field is exceptionally broad we will limit this to five interventions of which initial clinical and/or lab evaluations have suggested that they could be effective when implemented at a programmatic scale, and have been recommended by WHO for scale-up.

## Objectives

1. To identify, by systematic review of the literature, studies on evidence for scale-up for selected interventions in TB control.
2. To critically appraise and categorize these studies by:

- their objectives: evaluation of health outcomes (e.g. treatment outcomes, disease rates, adverse effects), delivery aspects or cost-effectiveness;
- their design, e.g. randomized comparison, non-randomized comparison, cohort study, cross sectional surveys;
- the setting in which the study was done: geographic (major regions), epidemiological (e.g. TB incidence, HIV prevalence) and research or clinical versus programmatic;
- their generalizability to settings beyond that in which the study was done;
- and for studies of health outcomes whether they primarily address efficacy or effectiveness.

1. identify, based on this appraisal, major research gaps in this type of evaluation.

The review focuses on research results that are accessible for international policy decisions and will therefore be restricted to reports that are in the public domain.

The following selected interventions will be subjected to review:

1. Isoniazid preventive therapy (IPT) for prevention of TB disease in HIV-infected individuals.
2. IPT for prevention of TB disease in household contacts of ingfectious TB patients.
3. Clinical algorithms for diagnosing smear-negative pulmonary TB (rule in)
4. Clinical algorithms for screening for pulmonary TB (rule out) in HIV-infected individuals
5. Methods for providing second-line TB treatment.

### **Design of the review**

### Literature sources

Publications will be selected from:

1. General databases (MEDLINE, EMBASE, Web of Science)
2. Regional databases (Index Medicus for the Eastern Mediterranean Region, SaudMed, INDMED, HERDIN, Thai Index Medicus, LILACS, African Index Medicus, Koreamed Medicus, Aidsthaidata)
3. The International Journal of TB and Lung Disease.

### Search plan

The initial search plan will aim at maximum inclusion of papers that contain descriptions of and data from the programmatic evaluation studies. It will therefore include:

- key word search of automated databases. See annex (Text S3) for search terms

General inclusion criteria for initial selection of articles include:

- Initially included will be all publications (scientific papers, reports, letters, reviews) that have the key words in title or abstract.
- Studies published between 01-01-1990 and 31-03-2012
- Publications written in other languages than English i.e. French, Spanish, German or Portuguese
- Studies from low and middle income countries

General exclusion criteria for initial selection of articles include:

- Publications with non-English abstracts
- Publications that appear not to address the selected interventions and/or an operational evaluation of these interventions
- Non-human studies
- Costing studies without an effectiveness component

Reviews will not be included but be kept apart for manual search.

Specific exclusion criteria for selection of articles include:

a. IPT for HIV-infected individuals or in household contacts

- Case reports
- Use in immunocompromised individuals other than HIV-infected
- Mathematical modeling studies not presenting observations on IPT

b. Clinical or screening algorithms

- Case reports
- Use in immunocompromised individuals other than HIV-infected
- Clinical studies limited to specific diagnostic tools (e.g. bronchoscopy, lung flute, PCR) and without addressing diagnostic algorithms

*NB we will include studies that evaluate various diagnostics in combination*

- TB prevalence studies/surveys that do not evaluate diagnostic methods or algorithms or do so without reference standard
- Proof-of-principle studies of diagnostic methods
- Studies on diagnostic tools that report sensitivity only
- Studies that only assess predictors of (smear-negative) TB without evaluating or developing an algorithm)
- Mathematical modeling studies not presenting observations on TB diagnosis

c. Second-line treatment

- Pharmacological studies
- Studies specifically aimed at assessing effects of drug resistance on treatment outcomes, including XDR
- Studies on resistance prevalence and patterns
- Diagnosis of drug-resistant TB
- Genetic studies
- Retrospective case series that report outcome data on less than 50 patients
- Studies on surgical intervention that only describe the patients who had surgery (*i.e. studies that describe a cohort of MDR patients of whom part had surgery are included*)
- Mathematical modeling studies not presenting observations on MDR treatment

Manual searches will be performed of reference lists of all retrieved review papers.

Additional manual search of the International Journal of Tuberculosis and Lung Disease will be done for a randomly selected 10% of the issues to check if the database search includes all relevant titles.

If not, a full manual search will be done of all issues.

All selected publications will be entered in an electronic database, and full text electronic or hard copies will be sought.

### Final study selection and data extraction

All included publications will read full text to check if all in- and exclusion criteria are met. Only full-text articles with complete description of objectives and study methodology will be included in the final selection.

Data will be extracted using the electronic database. Data to be extracted include:

- Characteristics of study setting: country (including TB, HIV and MDR burden), type and number of clinics, extent to which study setting is considered research or routine/programmatic. *See appraisal criteria.*
- Study objectives: summary of the study question, type of question addressed (health outcomes, delivery, cost-effectiveness). Intervention-specific:
  - IPT: effects on TB incidence, IPT completion, IPT adherence, side effects, other.
  - Diagnostic algorithms:
    - Evaluation of specified alogorithm, evaluation of distinct diagnostic procedures, evaluation of unspecified algorithm not of separate diagnostic procedures, improvement of smear examination, practices, additional diagnostics;
    - Generation of algorithm: no, yes without evaluation, yes with evaluation in training set only, yes with evaluation in prediction set.
  - Second-line treatment: treatment outcomes, treatment completion/adherence (with or without testing a specified hypothesis), side effects, surgery.
- Details of the study design: Prospective, retrospective or cross-sectional; comparative or non-comparative; if comparative: randomized or non-randomized (if randomized: group or individual; if group randomized: parallel or stepped-wedge); if non-randomized: parallel of before-after. Intervention-specific:
  - Diagnostic algorithms: use of reference standard; additional diagnostics assessed.
- Study population: numbers of participants, numbers with HIV infection, age distribution, specific limitations (e.g. children only, XDR patients only). Intervention-specific:
  - IPT: HIV-infected individuals or household contacts.
  - Diagnostic algorithms:
    - Rule in: TB suspects, smear-negative TB suspects, clinically diagnosed smear-negative TB cases.
    - Rule out: all HIV+ patients, patients eligible for TB screening according to program guidelines, patients eligible for TB screening according to study criteria, other.
  - Second-line treatment: high HIV prevalence in study population; proportion XDR.
- Generalizability: *see appraisal criteria*.
- Efficacy vs effectiveness: *see appraisal criteria.*

Free text will be allowed for comments on operational issues and feasibility if such data are available.

### Assessment of study quality

Assessment of quality will be limited to the description and categorization of the study design.

### Data synthesis

The extracted data will be described by the categorization mentioned in the objectives, and within this categorization, weighed by quality of the selected studies. Summary estimates (meta-analysis) of measurements/results will not be produced.

**Search terms**

See Text S3
