# Supplementary material for: Research on Implementation of Interventions in Tuberculosis Control in Low- and Middle-Income Countries: A Systematic Review
Source: PLoS Med. 2012 Dec 18;9(12):e1001358. doi: 10.1371/journal.pmed.1001358 (PMC3525528; doi:10.1371/journal.pmed.1001358)
Supplement: Text S3 — Search strategies, details of included studies, and full reference list. (DOCX) [file pmed.1001358.s003.docx]

**Text S3**

**Implementation research of interventions in tuberculosis control in low and middle income countries: a systematic review.**

*Frank Cobelens, Sanne van Kampen, Eleanor Ochodo, Rifat Atun, Christian Lienhardt*

**Summary of search strategies**

**Isoniazid Preventive Therapy**

in HIV-infected individuals and/or in household contacts

PubMed (http://www.ncbi.nlm.nih.gov/pubmed/)

(("isoniazid"[MeSH Terms] OR "isoniazid"[All Fields]) AND ("prevention and control"[Subheading] OR ("prevention"[All Fields] AND "control"[All Fields]) OR "prevention and control"[All Fields] OR ("preventive"[All Fields] AND "therapy"[All Fields]) OR "preventive therapy"[All Fields])) OR (("isoniazid"[MeSH Terms] OR "isoniazid"[All Fields]) AND ("prevention and control"[Subheading] OR ("prevention"[All Fields] AND "control"[All Fields]) OR "prevention and control"[All Fields] OR "prophylaxis"[All Fields]))

Species: Humans.

Web of Science (**http://www.webofknowledge.com/)**

TS=(Isoniazid prevent* OR Isoniazid prophylaxis OR Isoniazid chemoprophylaxis OR Isoniazid preventive therapy)

Databases=SCI-EXPANDED, SSCI, A&HCI.

EMBASE (http://www.embase.com/)

Isoniazid prevent* OR Isoniazid prophylaxis OR Isoniazid chemoprophylaxis OR Isoniazid preventive therapy

# Index Medicus for the Eastern Mediterranean Region (http://www.who.int/library/databases/emro)

Tuberculosis

SaudMed (<http://www.smj.org.sa/smjmedbase.asp>)

Tuberculosis

INDMED (http://indmed.nic.in/)

Isoniazid [Anywhere] OR Isoniazid prophylaxis [Anywhere] OR Isoniazid preventive therapy [Anywhere]

HERDIN (http://www.herdin.ph/)

Isoniazid [All fields] OR Isoniazid prophylaxis [All fields] OR Isoniazid chemoprophylaxis [All fields] OR Isoniazid preventive therapy [All fields]

Thai Index Medicus (http://library.md.chula.ac.th)

Isoniazid [All fields] OR Isoniazid prophylaxis [All fields] OR Isoniazid preventive therapy [All fields]

LILACS (http://bases.bireme.br)

"ISONIAZID" OR "ISONIAZIDA/" OR "ISONIAZIDE" [Words] or Isoniazid prophylaxis [Words] or Isoniazid preventive therapy [Words]

African Index Medicus (http://www.who.int/library/databases/afro/en/)

Tuberculosis

Koreamed Medicus (http://www.koreamed.org)

isoniazid [ALL] OR (tuberculosis [ALL] and preventive [ALL])

Aidsthaidata (http://www.aidsthaidata.org)

Tuberculosis

**Diagnostic algorithms of smear-negative pulmonary tuberculosis**

for “rule-in” or for “rule-out” diagnosis

PubMed (http://www.ncbi.nlm.nih.gov/pubmed/)

Diagnosis of smear negative pulmonary tuberculosis OR diagnosis of sputum negative pulmonary tuberculosis OR screening of smear negative pulmonary tuberculosis OR screening of sputum negative pulmonary tuberculosis OR diagnostic algorithm for smear negative pulmonary tuberculosis OR algorithm for tuberculosis screening OR algorithm for tuberculosis diagnosis OR case detection of smear negative pulmonary tuberculosis OR case finding of smear negative pulmonary tuberculosis

Species: Humans. Fields: Titles/Abstracts

Web of Science (**http://www.webofknowledge.com/)**

TS=(diagnosis of smear negative pulmonary tuberculosis OR diagnosis of sputum negative pulmonary tuberculosis OR screening of smear negative pulmonary tuberculosis OR screening of sputum negative pulmonary tuberculosis OR diagnostic algorithm for smear negative pulmonary tuberculosis OR algorithm for tuberculosis screening OR algorithm for tuberculosis diagnosis OR case detection of smear negative pulmonary tuberculosis OR case finding of smear negative pulmonary tuberculosis)

Databases=SCI-EXPANDED, SSCI, A&HCI.

EMBASE (http://www.embase.com/)

Diagnosis of smear negative pulmonary tuberculosis OR diagnosis of sputum negative pulmonary tuberculosis OR screening of smear negative pulmonary tuberculosis OR screening of sputum negative pulmonary tuberculosis OR diagnostic algorithm for smear negative pulmonary tuberculosis OR algorithm for tuberculosis screening OR algorithm for tuberculosis diagnosis OR case detection of smear negative pulmonary tuberculosis OR case finding of smear negative pulmonary tuberculosis

# Index Medicus for the Eastern Mediterranean Region (http://www.who.int/library/databases/emro)

Tuberculosis

SaudMed (<http://www.smj.org.sa/smjmedbase.asp>)

Tuberculosis

INDMED (http://indmed.nic.in/)

Tuberculosis [Anywhere] AND Negative [Anywhere] AND Diagnosis [Anywhere]

HERDIN (http://www.herdin.ph/)

Tuberculosis [All fields] AND Negative [All fields] AND Diagnosis [All fields]

Thai Index Medicus (http://library.md.chula.ac.th)

Tuberculosis [All fields] AND Negative [All fields] AND Diagnosis [All fields]

LILACS (http://bases.bireme.br)

"ISONIAZID" OR "ISONIAZIDA/" OR "ISONIAZIDE" [Words] or Isoniazid prophylaxis [Words] or Isoniazid preventive therapy [Words]

African Index Medicus (http://www.who.int/library/databases/afro/en/)

Tuberculosis

Koreamed Medicus (http://www.koreamed.org)

tuberculosis [ALL] and (HIV [ALL] or “tuberculosis diagnosis” or diagnosis [**limited to** Clinical Trial, Comparative Study, Evaluation Studies, Multicenter Study, Original Article, Randomized Controlled Trial, Validation Studies, English]

Aidsthaidata (http://www.aidsthaidata.org)

Tuberculosis

**Second-line treatment for multidrug-resistant tuberculosis**

PubMed (http://www.ncbi.nlm.nih.gov/pubmed/)

(“Tuberculosis"[All Fields] AND “treatment”[All Fields] AND (“second line”[All Fields] OR “second-line”[All Fields])) OR ("Microb Drug Resist"[Journal] OR "mdr"[All Fields]) AND tb[All Fields] AND ("therapy"[Subheading] OR "therapy"[All Fields] OR "treatment"[All Fields] OR "therapeutics"[MeSH Terms] OR "therapeutics"[All Fields])) OR (“xdr”[All Fields] AND ("tuberculosis"[MeSH Terms] OR "tuberculosis"[All Fields]) AND ("therapy"[Subheading] OR "therapy"[All Fields] OR "treatment"[All Fields] OR "therapeutics"[MeSH Terms] OR "therapeutics"[All Fields]))

Web of Science (**http://www.webofknowledge.com/)**

TS=(MDR TB treatment OR MDR TB therapy OR Multi drug resistant Tuberculosis Treatment OR Multi drug resistant Tuberculosis Therapy OR Tuberculosis second line treatment OR Mycobacterium tuberculosis second line treatment)

Databases=SCI-EXPANDED, SSCI, A&HCI.

EMBASE

MDR TB treatment OR MDR TB therapy OR Multi drug resistant Tuberculosis Treatment OR Multi drug resistant Tuberculosis Therapy OR Tuberculosis second line treatment OR Mycobacterium tuberculosis second line treatment

# Index Medicus for the Eastern Mediterranean Region (http://www.who.int/library/databases/emro)

Tuberculosis

SaudMed (<http://www.smj.org.sa/smjmedbase.asp>)

Tuberculosis

INDMED (http://indmed.nic.in/)

Tuberculosis [Anywhere] AND Resistance [Anywhere] OR Multidrug Resistance [Anywhere]

HERDIN (http://www.herdin.ph/)

Tuberculosis [All fields] AND Resistance [All fields] OR Multidrug Resistance [All fields] OR Multidrug Resistant [All fields]

Thai Index Medicus (http://library.md.chula.ac.th)

Tuberculosis [All fields] AND Resistance [All fields] OR Multidrug Resistance [All fields]

LILACS (http://bases.bireme.br)

Tuberculosis [Words] and Resistance [Words] or multi drug resistance [Words]

African Index Medicus (http://www.who.int/library/databases/afro/en/)

Tuberculosis

Koreamed Medicus (http://www.koreamed.org)

Tuberculosis [ALL] and multidrug [ALL]

Aidsthaidata (http://www.aidsthaidata.org)

Tuberculosis

**Timespan for all searches**

01/01/1990 – 31/03/2012

**Details of included papers**

**1. Studies included in review on isoniazid-preventive therapy**

| Year | First author (reference) | Location | | Main objective | Setting | Design | Aimed at measuring | Generalizability |
| --- | --- | --- | --- | --- | --- | --- | --- | --- |
| **In HIV-infected individuals** | | | | | | | | |
| 1993 | Pape [1] | Haiti | Effect on TB incidence | | Research | Comparative, individuals, parallel, randomized | Mixed | Irrespective |
| 1995 | Aisu [2] | Uganda | IPT completion/ adherence, costs | | Routine | Non-comparative | N/A | Similar setting |
| 1996 | Saenghirunvattana [3] | Thailand | Effect on TB incidence | | Research | Comparative, individuals, parallel, randomized | Mixed | Similar setting |
| 1997 | Ngamvithayapong  [4] | Thailand | IPT completion/ adherence | | Routine | Non-comparative | N/A | Similar setting |
| 1997 | Hawken [5] | Kenya | Effect on TB incidence; IPT adherence, side effects | | Research | Comparative, individuals, parallel, randomized | Efficacy | Irrespective |
| 1997 | Whalen [6] | Uganda | Effect on TB incidence; IPT completion/ adherence, side effects | | Research | Comparative, individuals, parallel, randomized | Efficacy | Irrespective |
| 1998 | Halsey [7] | Haiti | Effect on TB incidence; IPT completion/ adherence, side effects | | Research | Comparative, individuals, parallel, randomized | Efficacy | Irrespective |
| 1998 | Mwinga [8] | Zambia | Effect on TB incidence; IPT adherence, side effects | | Research | Comparative, individuals, parallel, randomized | Efficacy | Irrespective |
| 1999 | Haller [9] | Ivory Coast | Effect on TB incidence; IPT adherence, side effects | | Research | Comparative, individuals, parallel, randomized | Efficacy | Irrespective |
| 2000 | Bakari [10] | Tanzania | IPT completion/ adherence, side effects | | Routine | Non-comparative | Mixed | Not beyond |
| 2000 | Fitzgerald [11] | Haiti | Effect on TB incidence | | Research | Comparative, individuals, parallel, randomized | Efficacy | Irrespective |
| 2000 | Fitzgerald [12] | Haiti | Effect on TB incidence | | Mixed | Comparative, individuals, parallel, non-randomized | Mixed | Irrespective |
| 2000 | Gordin [13] | Various Americas | Effect on TB incidence and drug resistance, IPT completion, side-effects | | Research | Comparative, individuals, parallel, randomized | Mixed | Irrespective |
| 2001 | Fitzgerald [14] | Haiti | Effect on TB incidence | | Research | Comparative, individuals, parallel, randomized | Efficacy | Irrespective |
| 2001 | Piyaworawong [15] | Thailand | IPT completion/  Adherence | | Routine | Comparative, groups, before-after, non-randomized | N/A | Similar setting |
| 2001 | Quigley [16] | Zambia | Effect on TB incidence, IPT completion | | Research | Comparative, individuals, parallel, randomized | Efficacy | Irrespective |
| 2001 | de Pinho [17] | Brazil | Effect on TB incidence, IPT completion | | Research | Comparative, individuals, parallel, non-randomized | Mixed | Irrespective |
| 2002 | Alaei [18] | Iran | Effect on TB incidence, IPT completion | | Routine | Non-comparative | Effectiveness | Similar setting |
| 2002 | Lugada [19] | Uganda | IPT completion/ adherence, side-effects, cost-effectiveness | | Mixed | Non-comparative | Mixed | Similar setting |
| 2003 | Churchyard [20] | South Africa | Effect on TB incidence and drug resistance | | Mixed | Comparative, individuals, parallel, non-randomized | Mixed | Irrespective |
| 2005 | Grant [21] | South Africa | Effect on TB incidence, side effects | | Mixed | Comparative, individuals, staggered, non-randomized | Effectiveness | Irrespective |
| 2005 | Hiransuthikul [22] | Thailand | Effect on TB incidence | | Routine | Comparative, individuals, parallel, randomized | Mixed | Similar setting |
| 2005 | Hiransuthikul [23] | Thailand | Assessment of practices | | Routine | Non-comparative | N/A | Similar setting |
| 2005 | Rowe [24] | South Africa | IPT completion | | Routine | Non-comparative | N/A | Similar setting |
| 2006 | Lim [25] | Uganda | Effect on progression to AIDS | | Research | Comparative, individuals, parallel, randomized | Efficacy | Irrespective |
| 2006 | Mugisha [26] | Uganda | IPT completion/ adherence, side effects | | Routine | Non-comparative | Effectiveness | Similar setting |
| 2006 | Shrestha [27] | Uganda | Cost-effectiveness | | Routine | Comparative, groups, parallel, randomized | N/A | Similar setting |
| 2006 | Szakacs [28] | South Africa | IPT completion/adherence | | Routine | Non-comparative | N/A | Similar setting |
| 2006 | Zar [29] | South Africa | Effect on TB incidence, side effects | | Research | Comparative, individuals, parallel, randomized | Efficacy | Irrespective |
| 2006 | Prasad [30] | India | Effect on TB incidence, side effects | | Research | Comparative, individuals, parallel, randomized | Efficacy | Irrespective |
| 2007 | Golub [31] | Brazil | Effect on TB incidence | | Routine | Comparative, groups, staggered, non-randomized | Effectiveness | other |
| 2007 | Mohammed [32] | South Africa | Effect on TB incidence | | Research | Comparative, individuals, parallel, randomized | Efficacy | other |
| 2007 | Arbelaez [33] | Colombia | Effect on TB incidence, IPT completion, side-effects | | Routine | Comparative, individuals, before-after, non-randomized | Mixed | other |
| 2008 | Munseri [34] | Tanzania | IPT completion, side effects | | Mixed | Non-comparative | Mixed | other |
| 2009 | de Souza [35] | Brazil | Effect on TB incidence, IPT completion/ adherence, side-effects | | Research | Non-comparative | Mixed | other |
| 2009 | le Roux [36] | South Africa | IPT completion/adherence, side effects | | Research | Comparative, individuals, parallel, randomized | Efficacy | other |
| 2009 | Sutton [37] | Cambodia | Cost-effectiveness | | Routine | Non-comparative | N/a | Similar setting |
| 2009 | Golub [38] | South Africa | Effect on TB incidence, IPT completion | | Routine | Comparative, individuals, parallel, non-randomized | Effectiveness | Irrespective |
| 2009 | Gray [39] | South Africa | Side-effects | | Research | Comparative, individuals, parallel, non-randomized | Efficacy | Irrespective |
| 2010 | Tedla [40] | Botswana | Side-effects | | Routine | Non-comparative | Effectiveness | Irrespective |
| 2010 | Mosimaneotsile [41] | Botswana | Effect on TB incidence and drug resistance, IPT completion/adherence, side-effects, cost-effectiveness | | Routine | Non-comparative | Mixed | Similar setting |
| 2010 | Bark [42] | Uganda | IPT completion | | Mixed | Non-comparative | N/A | Similar setting |
| 2010 | Charalambous [43] | South Africa | Effect on mortality | | Routine | Non-comparative | Effectiveness | Irrespective |
| 2010 | Durovni [44] | Brazil | IPT completion, side-effects | | Routine | Non-comparative | Effectiveness | Irrespective |
| 2010 | Frigati [45] | South Africa | Effect on TB incidence | | Mixed | Non-comparative | Mixed | Irrespective |
| 2010 | Grant [46] | South Africa | Promotion of IPT uptake | | Routine | Non-comparative | N/A | Not assessed |
| 2010 | Grant [47] | South Africa | Side-effects | | Routine | Non-comparative | Effectiveness | Irrespective |
| 2010 | Gray [48] | South Africa | Side-effects | | Mixed | Comparative, individuals, parallel, non-randomized | Effectiveness | Irrespective |
| 2010 | Lester [49] | South Africa | Barriers to implementation | | Routine | Non-comparative | N/A | Similar setting |
| 2011 | Gust [50] | Botswana | IPT completion/ adherence | | Mixed | Non-comparative | N/A | other |
| 2011 | Samandari [51] | Botswana | Effect on TB incidence, mortality and drug resistance; side-effects | | Routine | Comparative, individuals, parallel, non-randomized | Mixed | Irrespective |
| 2011 | Saraceni [52] | Brazil | Implementation of IPT and TST screening | | Routine | Non-comparative | N/A | Not beyond |
| 2011 | Fenner [53] | Multicountry cohort Africa | Effect on TB incidence | | Mixed | Non-comparative | Effectiveness | Irrespective |
| 2011 | Kabali [54] | Tanzania | Effect on TB incidence and mortality | | Research | Non-comparative | Mixed | Irrespective |
| 2011 | Khongphatthanayothin [55] | Thailand | Effect on TB incidence and TST conversion; side-effects | | Mixed | Non-comparative | Effectiveness | Irrespective |
| 2011 | Mindachew [56] | Ethiopia | IPT adherence | | Routine | Non-comparative | N/A | Similar setting |
| 2011 | Madhi [57] | South Africa | Effect on TB incidence, mortality and drug resistance; side-effects | | Research | Comparative, individuals, parallel, randomized | Efficacy | Irrespective |
| 2011 | Martinson [58] | South Africa | Effect on TB incidence, mortality and drug resistance; side-effects; IPT completion/adherence | | Research | Comparative, individuals, parallel, randomized | Efficacy | Irrespective |
| 2012 | Namuwenge [59] | Uganda | IPT completion | | Routine | Non-comparative | N/A | Similar setting |

| **In household contacts** |
| --- |

| 2000 | Santacute [60] | Brazil | IPT completion, side-effects | Routine |  | Effectiveness | Irrespective |
| --- | --- | --- | --- | --- | --- | --- | --- |
| 2002 | Claessens [61] | Malawi | Other | Routine | Non-comparative | N/A | Irrespective |
| 2003 | Zachariah [62] | Malawi | Other | Routine | Comparative, individuals, parallel, non-randomized | N/A | Similar setting |
| 2006 | Marais [63] | South Africa | Effect on TB incidence, IPT completion/adherence, side-effects | Routine | Non-comparative | Effectiveness | Similar setting |
| 2006 | van Zyl [64] | South Africa | IPT completion | Routine | Comparative, individuals, parallel, non-randomized | N/A | Similar setting |
| 2006 | Véjar Mourgués [65] | Chile | IPT completion | Routine | Non-comparative | N/A | Not beyond |
| 2007 | Bowerman [66] | Taiwan | IPT completion | Routine | Non-comparative | N/a | Irrespective |
| 2009 | Banu Rekha [67] | India | Other | Routine | Non-comparative | N/a | Irrespective |
| 2010 | Aminzadeh [68] | Iran | IPT completion | Routine | Non-comparative | N/A | Similar setting |
| 2010 | Van Wyk [69] | South Africa | Other | Routine | Non-comparative | N/A | Not beyond |
| 2011 | Garie [70] | Ethiopia | Effect on TB incidence, IPT completion/adherence | Mixed | Non-comparative | Effectiveness | Similar setting |
| 2011 | Gomes [71] | Guinea-Bissau | IPT completion/adherence | Routine | Non-comparative | N/A | Similar setting |
| 2011 | Pothukuchi [72] | India | Enrolment into IPT | Routine | Non-comparative | N/A | Similar setting |
| 2011 | Van Wyk [73] | South Africa | Enrolment into IPT | Mixed | Non-comparative | N/A | Other |

*Comparative designs – individual: individuals are units of comparison; groups: groups are unit of comparison; parallel: parallel in time.*

*Irrespective: generalizable irrespective of setting where data were collected. Similar settings: generalizable to settings that are similar in terms of epidemiology and/or health care system. Not beyond: not generalizable beyond the country or setting in which study was done. Other: generalizability unclear (e.g. delivery studies in research settings).*

*N/A: not applicable. TST: tuberculin skin test.*

**2. Studies included in review on clinical algorithms for diagnosing smear-negative tuberculosis**

| Year | First author (reference) | Location | Main objective | Setting | Design | Aimed at measuring | Generalizability |
| --- | --- | --- | --- | --- | --- | --- | --- |

| **For rule-in of smear-negative tuberculosis** |
| --- |

| 1995 | Mohan [74] | India | Evaluation diagnostic procedures, development of algorithm | Research | Non-comparative | Mixed | Similar setting |
| --- | --- | --- | --- | --- | --- | --- | --- |
| 1995 | Parry [75] | Malawi | Improvement of sputum collection | Routine, 1-3 facilities | Non-comparative | N/A | Similar setting |
| 1997 | Harries [76] | Malawi | Evaluation predefined algorithm | Routine, 1-3 facilities | Non-comparative | Effectiveness | Similar setting |
| 1997 | Samb [77] | Tanzania, Burundi | Evaluation diagnostic procedures, development of algorithm | Research | Non-comparative | Efficacy | Similar setting |
| 1997 | Wilkinson [78] | South Africa | Evaluation diagnostic procedures | Routine, 1-3 facilities | Non-comparative | Effectiveness | Irrespective |
| 1998 | Harries [79] | Malawi | Evaluation predefined algorithm | Routine, 1-3 facilities | Non-comparative | Effectiveness | Similar setting |
| 1999 | Aris [80] | Tanzania | Evaluation diagnostic procedures, development of algorithm | Mixed | Non-comparative | Mixed | Similar setting |
| 1999 | Li [81] | China | Improvement of sputum collection | Routine, >3 facilities | Non-comparative | N/A | Irrespective |
| 2000 | Wilkinson [82] | South Africa | Evaluation predefined algorithm | Routine, 1-3 facilities | Non-comparative | Mixed | Similar setting |
| 2001 | Hargreaves [83] | Malawi | Evaluation predefined algorithm | Routine, 1-3 facilities | Non-comparative | Effectiveness | Similar setting |
| 2001 | Harries [84] | Malawi | Assessment of practices | Routine, >3 facilities | Non-comparative | N/A | Not beyond |
| 2001 | Hawken [85] | Kenya | Improvement of smear examination | Routine, 1-3 facilities | Non-comparative | N/A | Not beyond |
| 2001 | Tessema [86] | Ethiopia | Evaluation diagnostic procedures, development of algorithm | Routine, 1-3 facilities | Non-comparative | Effectiveness | Similar setting |
| 2002 | Bruchfeld [87] | Ethiopia | Evaluation diagnostic procedures, development of algorithm | Routine, 1-3 facilities | Non-comparative | Effectiveness | Similar setting |
| 2002 | van Rheenen [88] | Zambia | Evaluation predefined algorithm | Routine, 1-3 facilities | Non-comparative | Effectiveness | Similar setting |
| 2003 | Kivihya-Ndugga [89] | Kenya | Additional diagnostics, cost-effectiveness | Mixed | Non-comparative | Mixed | Similar setting |
| 2003 | van Cleeff [90] | Kenya | Additional diagnostics | Mixed | Non-comparative | Mixed | Similar setting |
| 2003 | Lambert [91] | Ethiopia | Improvement of smear examination | Routine, >3 facilities | Comapartive, groups, before-after, non-randomized | N/A | Not beyond |
| 2003 | Okutan [92] | Turkey | Improvement of sputum collection | Routine, 1-3 facilities | Non-comparative | N/A | Similar setting |
| 2004 | Apers [93] | Zimbabwe | Evaluation diagnostic procedures | Routine, 1-3 facilities | Non-comparative | Mixed | Similar setting |
| 2004 | Berggren [94] | Ethiopia | Evaluation diagnostic procedures | Routine, 1-3 facilities | Non-comparative | Effectiveness | Similar setting |
| 2006 | English [95] | South Africa | Evaluation predefined algorithm | Routine, 1-3 facilities | Non-comparative | Effectiveness | Similar setting |
| 2006 | Mello [96] | Brazil | Evaluation diagnostic procedures, development of algorithm, evaluation of this algorithm in a separate data set | Mixed | Non-comparative | Effectiveness | Similar setting |
| 2006 | Siddiqi [97] | Pakistan | Evaluation predefined algorithm | Routine, 1-3 facilities | Non-comparative | Mixed | Similar setting |
| 2006 | Wilson [98] | South Africa | Evaluation predefined algorithm | Routine, 1-3 facilities | Non-comparative | Mixed | Similar setting |
| 2007 | Saranchuk [99] | South Africa | Evaluation predefined algorithm | Routine, 1-3 facilities | Non-comparative | Mixed | Not beyond |
| 2008 | Maciel [100] | Brazil | Improvement of sputum collection | Mixed | Non-comparative | N/A | Similar setting |
| 2008 | Morse [101] | Botswana | Additional diagnostics | Routine, 1-3 facilities | Non-comparative | Mixed | Irrespective |
| 2008 | Soto [102] | Peru | Evaluation diagnostic procedures, development of algorithm | Routine, 1-3 facilities | Non-comparative | Mixed | Similar setting |
| 2008 | Weber [103] | Thailand | Assessment of practices | Routine, >3 facilities | Non-comparative | N/A | Irrespective |
| 2008 | Chang [104] | Hong Kong | Improvement of sputum collection | Routine, 1-3 facilities | Non-comparative | N/A | Irrespective |
| 2009 | Bell [105] | Malawi | Improvement of sputum collection | Mixed | Non-comparative | N/A | Not beyond |
| 2009 | Scherer [106] | Brazil | Additional diagnostics, cost-effectiveness | Routine, 1-3 facilities | Non-comparative | Effectiveness | Irrespective |
| 2010 | Lin [107] | Taiwan | Additional diagnostics | Mixed | Comparative, individuals, parallel, non-randomized | Mixed | Irrespective |
| 2010 | Kalawat [108] | India | Additional diagnostics | Mixed | Non-comparative | Efficacy | Other |
| 2010 | Oberhelman [109] | Peru | Additional diagnostics | Mixed | Non-comparative | Mixed | Irrespective |
| 2011 | Soto [110] | Peru | Evaluation predefined algorithm | Routine, 1-3 facilities | Non-comparative | Mixed | Irrespective |
| 2011 | Koole [111] | Cambodia | Evaluation predefined algorithm | Routine, >3 facilities | Non-comparative | Effectiveness | Irrespective |
| 2011 | Soto [112] | Peru | Evaluation predefined algorithm | Routine, 1-3 facilities | Non-comparative | Effectiveness | Similar setting |
| 2011 | Porskrog [113] | Guinee-Bissau | Additional diagnostics | Routine, >3 facilities | Non-comparative | Effectiveness | Similar setting |
| 2011 | Holtz [114] | South Africa | Evaluation predefined algorithm | Routine, 1-3 facilities | Comparative, individuals, before-after, non-randomized | Mixed | Irrespective |
| 2011 | Walley [115] | Uganda | Evaluation predefined algorithm | Routine, 1-3 facilities | Comparative, individuals, before-after, non-randomized | Effectiveness | Irrespective |
| 2011 | Wilson [116] | South Africa | Evaluation predefined algorithm | Routine, >3 facilities | Non-comparative | Mixed | Irrespective |
| 2012 | Alavi-Naini [117] | Iran | Evaluation diagnostic procedures, development of algorithm | Mixed | Non-comparative | Mixed | Similar setting |

| **For rule-out of smear-negative tuberculosis**  = |
| --- |

| 2003 | Mosimaneotsile [118] | Botswana | Evaluation predefined algorithm | Routine, >3 facilities | Non-comparative | Effectiveness | Similar setting |
| --- | --- | --- | --- | --- | --- | --- | --- |
| 2004 | Mohammed [119] | South Africa | Evaluation diagnostic procedures, development of algorithm | Mixed | Non-comparative | Efficacy | Irrespective |
| 2005 | Sanchez [120] | Brazil | Evaluation diagnostic procedures, development of algorithm | Routine, 1-3 facilities | Non-comparative | Effectiveness | Similar setting |
| 2006 | Day [121] | South Africa | Evaluation diagnostic procedures, development of algorithm | Routine, 1-3 facilities | Non-comparative | Mixed | Irrespective |
| 2008 | Ccheng [122] | Cambodia | Evaluation diagnostic procedures, development of algorithm | Routine, 1-3 facilities | Non-comparative | Mixed | Irrespective |
| 2009 | Lewis [123] | South Africa | Evaluation diagnostic procedures, development of algorithm | Mixed | Non-comparative | Effectiveness | Similar setting |
| 2009 | Ayles [124] | Zambia | Evaluation diagnostic procedures, development of algorithm | Population survey | Non-comparative | Mixed | Irrespective |
| 2009 | Monkongdee [125] | Thailand, Vietnam | Evaluation diagnostic procedures | Routine, >3 facilities | Non-comparative | Effectiveness | Irrespective |
| 2009 | Shah [126] | Ethiopia | Evaluation predefined algorithm | Routine, 1-3 facilities | Non-comparative | Effectiveness | Irrespective |
| 2009 | Tamhane [127] | Cambodia | Evaluation diagnostic procedures, development of algorithm | Routine, 1-3 facilities | Non-comparative | Mixed | Irrespective |
| 2009 | Were [128] | Uganda | Evaluation diagnostic procedures, development of algorithm | Routine, >3 facilities | Non-comparative | Effectiveness | Similar setting |
| 2009 | Lawn [129] | South Africa | Additional diagnostics | Routine, 1-3 facilities | Non-comparative | Mixed | Irrespective |
| 2010 | Agizew [130] | Botswana | Evaluation diagnostic procedures | Routine, >3 facilities | Non-comparative | Effectiveness | Similar setting |
| 2010 | Cain [131] | Cambodia, Thailand, Vietnam | Evaluation diagnostic procedures, development of algorithm | Routine, >3 facilities | Non-comparative | Mixed | Irrespective |
| 2010 | Corbett [132] | Zimbabwe | Evaluation diagnostic procedures, development of algorithm | Population survey | Non-comparative | Mixed | Irrespective |
| 2010 | Bassett [133] | South Africa | Evaluation diagnostic procedures, development of algorithm | Routine, 1-3 facilities | Non-comparative | Effectiveness | Irrespective |
| 2010 | Churchyard [134] | South Africa | Evaluation predefined algorithm | Routine, 1-3 facilities | Non-comparative | Effectiveness | Similar setting |
| 2011 | Nguyen [135] | Vietnam | Evaluation predefined algorithm | Routine, 1-3 facilities | Non-comparative | Mixed | Irrespective |
| 2012 | Rangaka [136] | South Africa | Evaluation diagnostic procedures, development of algorithm | Mixed | Non-comparative | Mixed | Irrespective |

*Comparative designs – individual: individuals are units of comparison; groups: groups are unit of comparison; parallel: parallel in time.*

*Irrespective: generalizable irrespective of setting where data were collected. Similar settings: generalizable to settings that are similar in terms of epidemiology and/or health care system. Not beyond: not generalizable beyond the country or setting in which study was done. Other: generalizability unclear (e.g. delivery studies in research settings).*

*N/A: not applicable.*

**3. Studies included in review on provision of second-line tuberculosis treatment**

| Year | First author (reference) | Location | Main objective | Setting | Design | Aimed at measuring | Generalizability |
| --- | --- | --- | --- | --- | --- | --- | --- |
| 1996 | Suo [137] | Taiwan | Treatment outcomes | Specialized clinic | Non-comparative | Efficacy | Irrespective |
| 1996 | Maranetra [138] | Thailand | Treatment outcomes | Specialized clinic | Non-comparative | Efficacy | Irrespective |
| 1996 | Mangunnegoro [139] | Indonesia | Treatment outcomes | Specialized clinic | Non-comparative | Efficacy | Irrespective |
| 1999 | Maranetra [140] | Thailand | Treatment outcomes | Specialized clinic | Non-comparative | Efficacy | Irrespective |
| 2000 | Yew [141] | China | Treatment outcomes | Specialized clinic | Non-comparative | Effectiveness | Irrespective |
| 2001 | Kim [142] | South Korea | Treatment outcomes | Specialized clinic | Non-comparative | Effectiveness | Irrespective |
| 2001 | Furin [143] | Peru | Side effects | Pilot | Non-comparative | Effectiveness | Irrespective |
| 2002 | Suarez [144] | Peru | Treatment outcomes, cost-effectiveness | Programmatic | Non-comparative | Effectiveness | Irrespective |
| 2003 | Tupasi [145] | Philippines | Treatment outcomes | Pilot | Non-comparative | Effectiveness | Irrespective |
| 2003 | Mitnick [146] | Peru | Treatment outcomes | Pilot | Non-comparative | Effectiveness | Irrespective |
| 2004 | Park [147] | South Korea | Treatment outcomes | Specialized clinic | Non-comparative | Effectiveness | Irrespective |
| 2004 | van Deun [148] | Bangladesh | Treatment outcomes | Pilot | Non-comparative | Effectiveness | Irrespective |
| 2004 | Palermo [149] | Argentina | Treatment outcomes | Specialized clinic | Non-comparative | Effectiveness | Irrespective |
| 2004 | Choi [150] | Peru | Drug ordering system | Pilot | Comparative, groups, parallel, non-randomized | N/A | Similar settings |
| 2004 | Vega [151] | Peru | Side effects | Pilot | Non-comparative | Effectiveness | Irrespective |
| 2004 | Nathanson [152] | Various | Side effects | Various | Non-comparative | Effectiveness | Irrespective |
| 2005 | Leimane [153] | Latvia | Treatment outcomes | Programmatic | Non-comparative | Effectiveness | Irrespective |
| 2005 | Olle-Goig [154] | Bolivia | Treatment outcomes | Specialized clinic | Non-comparative | Effectiveness | Similar settings |
| 2005 | Torun [155] | Turkey | Side effects | Specialized clinic | Non-comparative | Effectiveness | Irrespective |
| 2006 | Chiang [156] | Taiwan | Treatment outcomes | Programmatic | Non-comparative | Effectiveness | Irrespective |
| 2006 | Shin [157] | Russian Federation | Treatment outcomes | Pilot | Non-comparative | Effectiveness | Irrespective |
| 2006 | Tupasi [158] | Philippines | Treatment outcomes, cost-effectiveness | Pilot | Non-comparative | Effectiveness | Irrespective |
| 2006 | Nathanson [159] | Various | Treatment outcomes | Various | Non-comparative | Effectiveness | Irrespective |
| 2006 | Holtz [160] | South Africa | Risk factors non-completion/non-adherence | Programmatic | Comparative, individuals, parallel, non-randomized | N/A | Similar settings |
| 2006 | Chalco [161] | Peru | Role of nurses in treatment support | Pilot | Non-comparative | N/A | Similar settings |
| 2006 | Leimane [162] | Latvia | Implementation & coverage | Programmatic | Non-comparative | N/A | Similar settings |
| 2006 | Prasad [163] | India | Treatment outcomes, side effects | Specialized clinic | Non-comparative | Effectiveness | Irrespective |
| 2007 | Cox [164] | Uzbekistan | Treatment outcomes | Pilot | Non-comparative | Effectiveness | Irrespective |
| 2007 | Torun [165] | Turkey | Treatment outcomes | Specialized clinic | Non-comparative | Effectiveness | Irrespective |
| 2007 | Shin [166] | Russian Federation | Treatment outcomes, side effects | Pilot | Non-comparative | Effectiveness | Similar settings |
| 2007 | Thomas [167] | India | Treatment outcomes | Pilot | Non-comparative | Effectiveness | Similar settings |
| 2007 | Clark [168] | Turkey | Intervention to improve adherence | Specialized clinic | Comparative, individuals, parallel, randomized | N/A | Similar settings |
| 2007 | Acha [169] | Peru | Intervention to improve adherence | Pilot | Non-comparative | N/A | Similar settings |
| 2007 | Joshi [170] | India | Treatment outcomes, side effects | Specialized clinic | Non-comparative | Effectiveness | Irrespective |
| 2008 | Kwon [171] | South Korea | Treatment outcomes | Specialized clinic | Non-comparative | Effectiveness | Irrespective |
| 2008 | Kim [172] | South Korea | Treatment outcomes | Programmatic | Non-comparative | Effectiveness | Irrespective |
| 2008 | Shean [173] | South Africa | Treatment outcomes | Programmatic | Non-comparative | Effectiveness | Irrespective |
| 2008 | Mitnick [174] | Peru | Treatment outcomes | Pilot | Non-comparative | Effectiveness | Irrespective |
| 2008 | Franke [175] | Peru | Risk factors non-completion/non-adherence | Pilot | Non-comparative | N/A | Similar settings |
| 2009 | Karagoz [176] | Turkey | Treatment outcomes | Specialized clinic | Non-comparative | Effectiveness | Irrespective |
| 2009 | Jeon [177] | South Korea | Treatment outcomes | Specialized clinic | Non-comparative | Effectiveness | Irrespective |
| 2009 | O’Donnell [178] | South Africa | Treatment outcomes, HIV+ | Specialized clinic | Non-comparative | Effectiveness | Irrespective |
| 2009 | Seung [179] | South Africa | Treatment outcomes, HIV+ | Pilot | Non-comparative | Effectiveness | Irrespective |
| 2009 | Rao [180] | Pakistan | Treatment outcomes | Specialized clinic | Non-comparative | Effectiveness | Irrespective |
| 2009 | Malla [181] | Nepal | Treatment outcomes | Programmatic | Non-comparative | Effectiveness | Irrespective |
| 2009 | Tabarsi [182] | Iran | Treatment outcomes | Specialized clinic | Non-comparative | Effectiveness | Similar settings |
| 2009 | Singla [183] | India | Treatment outcomes | Pilot | Non-comparative | Effectiveness | Similar settings |
| 2009 | Kliiman [184] | Estonia | Treatment outcomes | Programmatic | Non-comparative | Effectiveness | Irrespective |
| 2009 | Siqueira [185] | Brazil | Treatment outcomes | Specialized clinic | Non-comparative | Effectiveness | Irrespective |
| 2010 | Bloss [186] | Latvia | Side effects | Programmatic | Non-comparative | Effectiveness | Irrespective |
| 2010 | Quelapio [187] | Philippines | Delivery intervention to improve treatment outcomes | Programmatic | Comparative, groups, before-after, non-randomized | Effectiveness | Similar settings |
| 2010 | Brust [188] | South Africa | Treatment outcomes, risk factors non-adherence, HIV+ | Programmatic | Non-comparative | Effectiveness | Similar settings |
| 2010 | Heller [189] | South Africa | Delivery intervention to improve treatment outcomes, HIV+ | Programmatic | Comparative, individual, before-after, non-randomized | Effectiveness | Similar settings |
| 2010 | Van Deun [190] | Bangladesh | Comaprison for treatment outcomes of different drug regimens; side effects | Programmatic | Comparative, individual, before-after, non-randomized | Effectiveness | Irrespective |
| 2010 | Shin [191] | Russian Federation | Treatment outcomes | Pilot | Non-comparative | Effectiveness | Irrespective |
| 2010 | Leimane [192] | Latvia | Treatment outcomes | Programmatic | Non-comparative | Effectiveness | Irrespective |
| 2010 | Kim [193] | South Korea | Treatment outcomes | Specialized clinics | Non-comparative | Effectiveness | Irrespective |
| 2011 | Kvasnovsky [194] | South Africa | Treatment outcomes, side effects, HIV+ | Programmatic | Non-comparative | Effectiveness | Irrespective |
| 2011 | Jeon [195] | South Korea | Treatment outcomes | Specialized clinic | Non-comparative | Effectiveness | Irrespective |
| 2011 | Tang [196] | China | Treatment outcomes | Specialized clinic | Non-comparative | Effectiveness | Similar settings |
| 2011 | Podewils [197] | Latvia | Treatment outcomes, side effects | Programmatic | Non-comparative | Effectiveness | Irrespective |
| 2011 | Liu [198] | China | Treatment outcomes | Specialized clinic | Non-comparative | Effectiveness | Similar settings |
| 2011 | Baghaei [199] | Iran | Side effects | Specialized clinic | Non-comparative | Effectiveness | Irrespective |
| 2011 | Lee [200] | South Korea | Treatment outcomes, side effects | Multiple specialized clinics | Non-comparative | Effectiveness | Irrespective |
| 2011 | Bonnet [201] | Georgia | Treatment outcomes, drug resistance amplification, re-infection | Pilot | Non-comparative | Effectiveness | Irrespective |
| 2011 | Chadha [202] | India | Enrolment into second-line treatment | Programmatic | Non-comparative | N/A | Similar settings |
| 2011 | Farley [203] | South Africa | Treatment outcomes, HIV+ | Programmatic | Non-comparative | Effectiveness | Irrespective |
| 2011 | Isaakidis [204] | India | Treatment outcomes, completion/adherence, immunological recovery, HIV+ | Pilot | Non-comparative | Effectiveness | Irrespective |
| 2011 | Kunawararak [205] | Thailand | Adherence; treatment outcomes | Programmatic | Comparative, individual, parallel, randomized | Mixed | Similar settings |
| 2012 | Alexy [206] | Peru | Comparison of methods for treatment outcome monitoring | Pilot | Non-comparative | N/A | Irrespective |
| 2012 | Loveday [207] | South Africa | Treatment outcomes, HIV+ | Specialized clinic versus programmatic | Comparative, groups, parallel, non-randomized | Effectiveness | Similar settings |
| 2012 | Palacios [208] | Peru | Treatment outcomes, side effects, HIV+ | Programmatic | Non-comparative | Effectiveness | Irrespective |

*Pilot: DOTS-Plus pilot project. Comparative designs – individual: individuals are units of comparison; groups: groups are unit of comparison; parallel: parallel in time. Irrespective: generalizable irrespective of setting where data were collected. Similar settings: generalizable to settings that are similar in terms of epidemiology and/or health care system. Not beyond: not generalizable beyond the country or setting in which study was done. Other: generalizability unclear (e.g. delivery studies in research settings).*

*N/A: not applicable.*

*HIV+: patient population with high HIV prevalence*

**REFERENCES**

All reviewed publications

1. Pape JW, Jean SS, Ho JL, Hafner A, Johnson WD Jr (1993) Effect of isoniazid prophylaxis on incidence of active tuberculosis and progression of HIV infection. Lancet 342: 268–272.

2. Aisu T, Raviglione MC, van Praag E, Eriki P, Narain JP, et al. (1995) Preventive chemotherapy for HIV-associated tuberculosis in Uganda: an operational assessment at a voluntary counselling and testing centre. AIDS 9: 267–273.

3. Saenghirunvattana S (1996) Effect of isoniazid prophylaxis on incidence of active tuberculosis among Thai HIV-infected individuals. J Med Assoc Thai 79: 285–287.

4. Ngamvithayapong J, Uthaivoravit W, Yanai H, Akarasewi P, Sawanpanyalert P (1997) Adherence to tuberculosis preventive therapy among HIV-infected persons in Chiang Rai, Thailand. AIDS 11: 107–112.

5. Hawken MP, Meme HK, Elliott LC, Chakaya JM, Morris JS, et al. (1997) Isoniazid preventive therapy for tuberculosis in HIV-1-infected adults: results of a randomized controlled trial. AIDS 11: 875–882.

6. Whalen CC, Johnson JL, Okwera A, Hom DL, Huebner R, et al. (1997) A trial of three regimens to prevent tuberculosis in Ugandan adults infected with the human immunodeficiency virus. Uganda-Case Western Reserve University Research Collaboration. N Engl J Med 337: 801–808. doi:10.1056/NEJM199709183371201

7. Halsey NA, Coberly JS, Desormeaux J, Losikoff P, Atkinson J, et al. (1998) Randomised trial of isoniazid versus rifampicin and pyrazinamide for prevention of tuberculosis in HIV-1 infection. Lancet 351: 786–792. doi:10.1016/S0140-6736(97)06532-X

8. Mwinga A, Hosp M, Godfrey-Faussett P, Quigley M, Mwaba P, et al. (1998) Twice weekly tuberculosis preventive therapy in HIV infection in Zambia. AIDS 12: 2447–2457.

9. Haller L, Sossouhounto R, Coulibaly IM, Dosso M, Kone M, et al. (1999) Isoniazid plus sulphadoxine-pyrimethamine can reduce morbidity of HIV-positive patients treated for tuberculosis in Africa: a controlled clinical trial. Chemotherapy 45: 452–465.

10. Bakari M, Moshi A, Aris EA, Chale S, Josiah R, et al. (2000) Isoniazid prophylaxis for tuberculosis prevention among HIV infected police officers in Dar es Salaam. East Afr Med J 77: 494–497.

11. Fitzgerald DW, Severe P, Joseph P, Mellon LR, Noel E, et al. (2001) No effect of isoniazid prophylaxis for purified protein derivative-negative HIV-infected adults living in a country with endemic tuberculosis: results of a randomized trial. J Acquir Immune Defic Syndr 28: 305–307.

12. Fitzgerald DW, Morse MM, Pape JW, Johnson WD (2000) Active tuberculosis in individuals infected with human immunodeficiency virus after isoniazid prophylaxis. Clin Infect Dis 31: 1495–1497. doi:10.1086/317485

13. Gordin F, Chaisson RE, Matts JP, Miller C, de Lourdes Garcia M, et al. (2000) Rifampin and pyrazinamide vs isoniazid for prevention of tuberculosis in HIV-infected persons: an international randomized trial. Terry Beirn Community Programs for Clinical Research on AIDS, the Adult AIDS Clinical Trials Group, the Pan American Health Organization, and the Centers for Disease Control and Prevention Study Group. JAMA 283: 1445–1450.

14. Fitzgerald DW, Desvarieux M, Severe P, Joseph P, Johnson WD, et al. (2000) Effect of post-treatment isoniazid on prevention of recurrent tuberculosis in HIV-1-infected individuals: a randomised trial. Lancet 356: 1470–1474. doi:10.1016/S0140-6736(00)02870-1

15. Piyaworawong S, Yanai H, Nedsuwan S, Akarasewi P, Moolphate S, et al. (2001) Tuberculosis preventative therapy as part of a care package for people living with HIV in a district of Thailand. AIDS 15: 1739–1741.

16. Quigley MA, Mwinga A, Hosp M, Lisse I, Fuchs D, et al. (2001) Long-term effect of preventive therapy for tuberculosis in a cohort of HIV-infected Zambian adults. AIDS 15: 215–222.

17. de Pinho AM, Santoro-Lopes G, Harrison LH, Schechter M (2001) Chemoprophylaxis for tuberculosis and survival of HIV-infected patients in Brazil. AIDS 15: 2129–2135.

18. Alaei K, Alaei A, Mansouri D (2002) Reduction of clinical tuberculosis in HIV-infected males with isoniazid prophylaxis. East Mediterr Health J 8: 754–757.

19. Lugada ES, Watera C, Nakiyingi J, Elliott A, Brink A, et al. (2002) Operational assessment of isoniazid prophylaxis in a community AIDS service organisation in Uganda. Int J Tuberc Lung Dis 6: 326–331.

20. Churchyard GJ, Fielding K, Charalambous S, Day JH, Corbett EL, et al. (2003) Efficacy of secondary isoniazid preventive therapy among HIV-infected Southern Africans: time to change policy? AIDS 17: 2063–2070. doi:10.1097/01.aids.0000076319.42412.70

21. Grant AD, Charalambous S, Fielding KL, Day JH, Corbett EL, et al. (2005) Effect of routine isoniazid preventive therapy on tuberculosis incidence among HIV-infected men in South Africa: a novel randomized incremental recruitment study. JAMA 293: 2719–2725. doi:10.1001/jama.293.22.2719

22. Hiransuthikul N, Nelson KE, Hiransuthikul P, Vorayingyong A, Paewplot R (2005) INH preventive therapy among adult HIV-infected patients in Thailand. Int J Tuberc Lung Dis 9: 270–275.

23. Hiransuthikul N, Hiransuthikul P, Nelson KE, Jirawisit M, Paewplot R, et al. (2005) Physician adherence to isoniazid preventive therapy guidelines for HIV-infected patients in Thailand. Southeast Asian J Trop Med Public Health 36: 1208–1215.

24. Rowe KA, Makhubele B, Hargreaves JR, Porter JD, Hausler HP, et al. (2005) Adherence to TB preventive therapy for HIV-positive patients in rural South Africa: implications for antiretroviral delivery in resource-poor settings? Int J Tuberc Lung Dis 9: 263–269.

25. Lim HJ, Okwera A, Mayanja-Kizza H, Ellner JJ, Mugerwa RD, et al. (2006) Effect of tuberculosis preventive therapy on HIV disease progression and survival in HIV-infected adults. HIV Clin Trials 7: 172–183. doi:10.1310/hct0704-172

26. Mugisha B, Bock N, Mermin J, Odeke RM, Miller B, et al. (2006) Tuberculosis case finding and preventive therapy in an HIV voluntary counseling and testing center in Uganda. Int J Tuberc Lung Dis 10: 761–767.

27. Shrestha RK, Mugisha B, Bunnell R, Mermin J, Hitimana-Lukanika C, et al. (2006) Cost-effectiveness of including tuberculin skin testing in an IPT program for HIV-infected persons in Uganda. Int J Tuberc Lung Dis 10: 656–662.

28. Szakacs TA, Wilson D, Cameron DW, Clark M, Kocheleff P, et al. (2006) Adherence with isoniazid for prevention of tuberculosis among HIV-infected adults in South Africa. BMC Infect Dis 6: 97. doi:10.1186/1471-2334-6-97

29. Zar HJ, Cotton MF, Strauss S, Karpakis J, Hussey G, et al. (2007) Effect of isoniazid prophylaxis on mortality and incidence of tuberculosis in children with HIV: randomised controlled trial. BMJ 334: 136. doi:10.1136/bmj.39000.486400.55

30. Prasad B (2006) A study on comparative efficacy of anti tubercular chemoprophylaxis with 2 different regimens: 2 months of rifampicin and pyrazinamide and 6 months of daily isoniazid and rifampicin in HIV infected tuberculin reactors. Lung India 23: 64. doi:10.4103/0970-2113.44411

31. Golub JE, Saraceni V, Cavalcante SC, Pacheco AG, Moulton LH, et al. (2007) The impact of antiretroviral therapy and isoniazid preventive therapy on tuberculosis incidence in HIV-infected patients in Rio de Janeiro, Brazil. AIDS 21: 1441–1448. doi:10.1097/QAD.0b013e328216f441

32. Mohammed A, Myer L, Ehrlich R, Wood R, Cilliers F, et al. (2007) Randomised controlled trial of isoniazid preventive therapy in South African adults with advanced HIV disease. Int J Tuberc Lung Dis 11: 1114–1120.

33. Arbeláez MP, Arbeláez A, Gómez RD, Rojas C, Vélez L, et al. (2007) [Effectiveness of prophylaxis against tuberculosis in patients infected with HIV]. Biomedica 27: 515–525.

34. Munseri PJ, Talbot EA, Mtei L, Fordham von Reyn C (2008) Completion of isoniazid preventive therapy among HIV-infected patients in Tanzania. Int J Tuberc Lung Dis 12: 1037–1041.

35. Souza CTV de, Hökerberg YHM, Pacheco SJB, Rolla VC, Passos SRL (2009) Effectiveness and safety of isoniazid chemoprophylaxis for HIV-1 infected patients from Rio de Janeiro. Mem Inst Oswaldo Cruz 104: 462–467.

36. le Roux SM, Cotton MF, Golub JE, le Roux DM, Workman L, et al. (2009) Adherence to isoniazid prophylaxis among HIV-infected children: a randomized controlled trial comparing two dosing schedules. BMC Med 7: 67. doi:10.1186/1741-7015-7-67

37. Sutton BS, Arias MS, Chheng P, Eang MT, Kimerling ME (2009) The cost of intensified case finding and isoniazid preventive therapy for HIV-infected patients in Battambang, Cambodia. Int J Tuberc Lung Dis 13: 713–718.

38. Golub JE, Pronyk P, Mohapi L, Thsabangu N, Moshabela M, et al. (2009) Isoniazid preventive therapy, HAART and tuberculosis risk in HIV-infected adults in South Africa: a prospective cohort. AIDS 23: 631–636. doi:10.1097/QAD.0b013e328327964f

39. Gray D, Nuttall J, Lombard C, Davies M-A, Workman L, et al. (2009) Low Rates of Hepatotoxicity in HIV-infected Children on Anti-retroviral Therapy with and Without Isoniazid Prophylaxis. J Trop Pediatr. Available: http://www.ncbi.nlm.nih.gov/pubmed/19710246.

40. Tedla Z, Nyirenda S, Peeler C, Agizew T, Sibanda T, et al. (2010) Isoniazid-associated hepatitis and antiretroviral drugs during tuberculosis prophylaxis in hiv-infected adults in Botswana. Am. J. Respir. Crit. Care Med 182: 278–285. doi:10.1164/rccm.200911-1783OC

41. Mosimaneotsile B, Mathoma A, Chengeta B, Nyirenda S, Agizew TB, et al. (2009) Isoniazid Tuberculosis Preventive Therapy in HIV-Infected Adults Accessing Antiretroviral Therapy: A Botswana Experience, 2004-2006. J Acquir Immune Defic Syndr. Available: http://www.ncbi.nlm.nih.gov/pubmed/19934764.

42. Bark CM, Morrison CS, Salata RA, Byamugisha JK, Katalemwa NH, et al. (2010) Acceptability of treatment of latent tuberculosis infection in newly HIV-infected young women in Uganda. Int J Tuberc Lung Dis 14: 1647–1649.

43. Charalambous S, Grant AD, Innes C, Hoffmann CJ, Dowdeswell R, et al. (2010) Association of isoniazid preventive therapy with lower early mortality in individuals on antiretroviral therapy in a workplace programme. AIDS 24 Suppl 5: S5–13. doi:10.1097/01.aids.0000391010.02774.6f

44. Durovni B, Cavalcante SC, Saraceni V, Vellozo V, Israel G, et al. (2010) The implementation of isoniazid preventive therapy in HIV clinics: the experience from the TB/HIV in Rio (THRio) study. AIDS 24 Suppl 5: S49–56. doi:10.1097/01.aids.0000391022.95412.a6

45. Frigati LJ, Kranzer K, Cotton MF, Schaaf HS, Lombard CJ, et al. (2011) The impact of isoniazid preventive therapy and antiretroviral therapy on tuberculosis in children infected with HIV in a high tuberculosis incidence setting. Thorax 66: 496–501. doi:10.1136/thx.2010.156752

46. Grant AD, Coetzee L, Fielding KL, Lewis JJ, Ntshele S, et al. (2010) “Team up against TB”: promoting involvement in Thibela TB, a trial of community-wide tuberculosis preventive therapy. AIDS 24 Suppl 5: S37–44. doi:10.1097/01.aids.0000391020.10661.eb

47. Grant AD, Mngadi KT, van Halsema CL, Luttig MM, Fielding KL, et al. (2010) Adverse events with isoniazid preventive therapy: experience from a large trial. AIDS 24 Suppl 5: S29–36. doi:10.1097/01.aids.0000391019.10661.66

48. Gray D, Nuttall J, Lombard C, Davies M, Workman L, et al. (2010) Low rates of hepatotoxicity in HIV-infected children on anti-retroviral therapy with and without isoniazid prophylaxis. J Trop. Pediatr 56: 159–165. doi:10.1093/tropej/fmp079

49. Lester R, Hamilton R, Charalambous S, Dwadwa T, Chandler C, et al. (2010) Barriers to implementation of isoniazid preventive therapy in HIV clinics: a qualitative study. AIDS 24 Suppl 5: S45–48. doi:10.1097/01.aids.0000391021.18284.12

50. Gust DA, Mosimaneotsile B, Mathebula U, Chingapane B, Gaul Z, et al. (2011) Risk factors for non-adherence and loss to follow-up in a three-year clinical trial in Botswana. PLoS ONE 6: e18435. doi:10.1371/journal.pone.0018435

51. Samandari T, Agizew TB, Nyirenda S, Tedla Z, Sibanda T, et al. (2011) 6-month versus 36-month isoniazid preventive treatment for tuberculosis in adults with HIV infection in Botswana: a randomised, double-blind, placebo-controlled trial. Lancet 377: 1588–1598. doi:10.1016/S0140-6736(11)60204-3

52. Saraceni V, Pacheco AG, Golub JE, Vellozo V, King BS, et al. (2011) Physician adherence to guidelines for tuberculosis and HIV care in Rio de Janeiro, Brazil. Braz J Infect Dis 15: 249–252.

53. Fenner L, Forster M, Boulle A, Phiri S, Braitstein P, et al. (2011) Tuberculosis in HIV programmes in lower-income countries: practices and risk factors. Int J Tuberc Lung Dis 15: 620–627. doi:10.5588/ijtld.10.0249

54. Kabali C, von Reyn CF, Brooks DR, Waddell R, Mtei L, et al. (2011) Completion of isoniazid preventive therapy and survival in HIV-infected, TST-positive adults in Tanzania. Int J Tuberc Lung Dis 15: 1515–1521, i. doi:10.5588/ijtld.10.0788

55. Khongphatthanayothin M, Avihingsanon A, Teeratakulpisarn N, Phanuphak N, Buajoom R, et al. (2012) Feasibility and efficacy of isoniazid prophylaxis for latent tuberculosis in HIV-infected clients patients in Thailand. AIDS Res Hum Retroviruses 28: 270–275. doi:10.1089/aid.2011.0041

56. Mindachew M, Deribew A, Tessema F, Biadgilign S (2011) Predictors of adherence to isoniazid preventive therapy among HIV positive adults in Addis Ababa, Ethiopia. BMC Public Health 11: 916. doi:10.1186/1471-2458-11-916

57. Madhi SA, Nachman S, Violari A, Kim S, Cotton MF, et al. (2011) Primary isoniazid prophylaxis against tuberculosis in HIV-exposed children. N Engl J Med 365: 21–31. doi:10.1056/NEJMoa1011214

58. Martinson NA, Barnes GL, Moulton LH, Msandiwa R, Hausler H, et al. (2011) New regimens to prevent tuberculosis in adults with HIV infection. N Engl J Med 365: 11–20. doi:10.1056/NEJMoa1005136

59. Namuwenge PM, Mukonzo JK, Kiwanuka N, Wanyenze R, Byaruhanga R, et al. (2012) Loss to follow up from isoniazid preventive therapy among adults attending HIV voluntary counseling and testing sites in Uganda. Trans R Soc Trop Med Hyg 106: 84–89. doi:10.1016/j.trstmh.2011.10.015

60. Santacute;Anna CC, David SG, Marques AM (2000) [Antituberculosis chemoprophylaxis in a public hospital - study of 100 children]. J Pediatr (Rio J) 76: 413–420.

61. Santacute;Anna CC, David SG, Marques AM (2000) [Antituberculosis chemoprophylaxis in a public hospital - study of 100 children]. J Pediatr (Rio J) 76: 413–420.

62. Zachariah R, Spielmann MP, Harries AD, Gomani P, Graham SM, et al. (2003) Passive versus active tuberculosis case finding and isoniazid preventive therapy among household contacts in a rural district of Malawi. Int J Tuberc Lung Dis 7: 1033–1039.

63. Marais BJ, van Zyl S, Schaaf HS, van Aardt M, Gie RP, et al. (2006) Adherence to isoniazid preventive chemotherapy: a prospective community based study. Arch Dis Child 91: 762–765. doi:10.1136/adc.2006.097220

64. van Zyl S, Marais BJ, Hesseling AC, Gie RP, Beyers N, et al. (2006) Adherence to anti-tuberculosis chemoprophylaxis and treatment in children. Int J Tuberc Lung Dis 10: 13–18.

65. Véjar Mourgués L, Henao R M (2006) Adherencia a quimioprofilaxis de niños chilenos expuestos a tuberculosis del adulto. Revista chilena de enfermedades respiratorias 22. Available: http://www.scielo.cl/scielo.php?script=sci_isoref&pid=S0717-73482006000300003&lng=es.

66. Bowerman RJ (2007) Community-wide INH treatment of latent TB infection in a BCG-vaccinated population: experience in rural Taiwan. Int J Tuberc Lung Dis 11: 470–472.

67. Banu Rekha VV, Jagarajamma K, Wares F, Chandrasekaran V, Swaminathan S (2009) Contact screening and chemoprophylaxis in India’s Revised Tuberculosis Control Programme: a situational analysis. Int J Tuberc Lung Dis 13: 1507–1512.

68. Aminzadeh Z, Asl RT (2011) A six months follow-up on children less than 6 years old in contact with smear positive tuberculosis patients, varamin city, tehran, iran. Int J Prev Med 2: 79–81.

69. Van Wyk SS, Hamade H, Hesseling AC, Beyers N, Enarson DA, et al. (2010) Recording isoniazid preventive therapy delivery to children: operational challenges. Int J Tuberc Lung Dis 14: 650–653.

70. Garie KT, Yassin MA, Cuevas LE (2011) Lack of adherence to isoniazid chemoprophylaxis in children in contact with adults with tuberculosis in Southern Ethiopia. PLoS ONE 6: e26452. doi:10.1371/journal.pone.0026452

71. Gomes VF, Wejse C, Oliveira I, Andersen A, Vieira FJ, et al. (2011) Adherence to isoniazid preventive therapy in children exposed to tuberculosis: a prospective study from Guinea-Bissau. Int J Tuberc Lung Dis 15: 1637–1643. doi:10.5588/ijtld.10.0558

72. Pothukuchi M, Nagaraja SB, Kelamane S, Satyanarayana S, Shashidhar, et al. (2011) Tuberculosis contact screening and isoniazid preventive therapy in a South Indian district: operational issues for programmatic consideration. PLoS ONE 6: e22500. doi:10.1371/journal.pone.0022500

73. van Wyk SS, Reid AJ, Mandalakas AM, Enarson DA, Beyers N, et al. (2011) Operational challenges in managing Isoniazid Preventive Therapy in child contacts: a high-burden setting perspective. BMC Public Health 11: 544. doi:10.1186/1471-2458-11-544

74. Mohan A, Pande JN, Sharma SK, Rattan A, Guleria R, et al. (1995) Bronchoalveolar lavage in pulmonary tuberculosis: a decision analysis approach. QJM 88: 269–276.

75. Parry CM, Kamoto O, Harries AD, Wirima JJ, Nyirenda CM, et al. (1995) The use of sputum induction for establishing a diagnosis in patients with suspected pulmonary tuberculosis in Malawi. Tuber Lung Dis 76: 72–76.

76. Harries AD, Kamenya A, Subramanyam VR, Maher D, Squire SB, et al. (1997) Screening pulmonary tuberculosis suspects in Malawi: testing different strategies. Trans R Soc Trop Med Hyg 91: 416–419.

77. Samb B, Henzel D, Daley CL, Mugusi F, Niyongabo T, et al. (1997) Methods for diagnosing tuberculosis among in-patients in eastern Africa whose sputum smears are negative. Int J Tuberc Lung Dis 1: 25–30.

78. Wilkinson D, De Cock KM, Sturm AW (1997) Diagnosing tuberculosis in a resource-poor setting: the value of a trial of antibiotics. Trans R Soc Trop Med Hyg 91: 422–424.

79. Harries AD, Banda HT, Boeree MJ, Welby S, Wirima JJ, et al. (1998) Management of pulmonary tuberculosis suspects with negative sputum smears and normal or minimally abnormal chest radiographs in resource-poor settings. Int J Tuberc Lung Dis 2: 999–1004.

80. Aris EA, Bakari M, Chonde TM, Kitinya J, Swai AB (1999) Diagnosis of tuberculosis in sputum negative patients in Dar es Salaam. East Afr Med J 76: 630–634.

81. Li LM, Bai LQ, Yang HL, Xiao CF, Tang RY, et al. (1999) Sputum induction to improve the diagnostic yield in patients with suspected pulmonary tuberculosis. Int J Tuberc Lung Dis 3: 1137–1139.

82. Wilkinson D, Newman W, Reid A, Squire SB, Sturm AW, et al. (2000) Trial-of-antibiotic algorithm for the diagnosis of tuberculosis in a district hospital in a developing country with high HIV prevalence. Int J Tuberc Lung Dis 4: 513–518.

83. Hargreaves NJ, Kadzakumanja O, Phiri S, Nyangulu DS, Salaniponi FM, et al. (2001) What causes smear-negative pulmonary tuberculosis in Malawi, an area of high HIV seroprevalence? Int J Tuberc Lung Dis 5: 113–122.

84. Harries AD, Hargreaves NJ, Kwanjana JH, Salaniponi FM (2001) Clinical diagnosis of smear-negative pulmonary tuberculosis: an audit of diagnostic practice in hospitals in Malawi. Int J Tuberc Lung Dis 5: 1143–1147.

85. Hawken MP, Muhindi DW, Chakaya JM, Bhatt SM, Ng’ang’a LW, et al. (2001) Under-diagnosis of smear-positive pulmonary tuberculosis in Nairobi, Kenya. Int J Tuberc Lung Dis 5: 360–363.

86. Tessema TA, Bjune G, Assefa G, Bjorvat B (2001) An evaluation of the diagnostic value of clinical and radiological manifestations in patients attending the addis ababa tuberculosis centre. Scand J Infect Dis 33: 355–361.

87. Bruchfeld J, Aderaye G, Palme IB, Bjorvatn B, Britton S, et al. (2002) Evaluation of outpatients with suspected pulmonary tuberculosis in a high HIV prevalence setting in Ethiopia: clinical, diagnostic and epidemiological characteristics. Scand J Infect Dis 34: 331–337.

88. Van Rheenen P (2002) The use of the paediatric tuberculosis score chart in an HIV-endemic area. Trop Med Int Health 7: 435–441.

89. Kivihya-Ndugga LEA, van Cleeff MRA, Githui WA, Nganga LW, Kibuga DK, et al. (2003) A comprehensive comparison of Ziehl-Neelsen and fluorescence microscopy for the diagnosis of tuberculosis in a resource-poor urban setting. Int J Tuberc Lung Dis 7: 1163–1171.

90. van Cleeff MRA, Kivihya-Ndugga L, Githui W, Nganga L, Odhiambo J, et al. (2003) A comprehensive study of the efficiency of the routine pulmonary tuberculosis diagnostic process in Nairobi. Int J Tuberc Lung Dis 7: 186–189.

91. Lambert ML, Sugulle H, Seyoum D, Abdurahman S, Abdinasir A, et al. (2003) How can detection of infectious tuberculosis be improved? Experience in the Somali region of Ethiopia. Int J Tuberc Lung Dis 7: 485–488.

92. Okutan O, Kartaloglu Z, Kilic E, Bozkanat E, Ilvan A (2003) Diagnostic contribution of gastric and bronchial lavage examinations in cases suggestive of pulmonary tuberculosis. Yonsei Med J 44: 242–248.

93. Apers L, Wijarajah C, Mutsvangwa J, Chigara N, Mason P, et al. (2004) Accuracy of routine diagnosis of pulmonary tuberculosis in an area of high HIV prevalence. Int J Tuberc Lung Dis 8: 945–951.

94. Berggren Palme I, Gudetta B, Bruchfeld J, Eriksson M, Giesecke J (2004) Detection of Mycobacterium tuberculosis in gastric aspirate and sputum collected from Ethiopian HIV-positive and HIV-negative children in a mixed in- and outpatient setting. Acta Paediatr 93: 311–315.

95. English RG, Bachmann MO, Bateman ED, Zwarenstein MF, Fairall LR, et al. (2006) Diagnostic accuracy of an integrated respiratory guideline in identifying patients with respiratory symptoms requiring screening for pulmonary tuberculosis: a cross-sectional study. BMC Pulm Med 6: 22. doi:10.1186/1471-2466-6-22

96. Mello FC de Q, Bastos LG do V, Soares SLM, Rezende VMC, Conde MB, et al. (2006) Predicting smear negative pulmonary tuberculosis with classification trees and logistic regression: a cross-sectional study. BMC Public Health 6: 43. doi:10.1186/1471-2458-6-43

97. Siddiqi K, Walley J, Khan MA, Shah K, Safdar N (2006) Clinical guidelines to diagnose smear-negative pulmonary tuberculosis in Pakistan, a country with low-HIV prevalence. Trop Med Int Health 11: 323–331. doi:10.1111/j.1365-3156.2006.01559.x

98. Wilson D, Nachega J, Morroni C, Chaisson R, Maartens G (2006) Diagnosing smear-negative tuberculosis using case definitions and treatment response in HIV-infected adults. Int J Tuberc Lung Dis 10: 31–38.

99. Saranchuk P, Boulle A, Hilderbrand K, Coetzee D, Bedelu M, et al. (2007) Evaluation of a diagnostic algorithm for smear-negative pulmonary tuberculosis in HIV-infected adults. S Afr Med J 97: 517–523.

100. Maciel ELN, Dietze R, Silva REC de F, Hadad DJ, Struchiner CJ (2008) Avaliação do sistema de pontuação para o diagnóstico da tuberculose na infância preconizado pelo Ministério da Saúde, Brasil. Cadernos de Saúde Pública 24: 402–408. doi:10.1590/S0102-311X2008000200019

101. Morse M, Kessler J, Albrecht S, Kim R, Thakur R, et al. (2008) Induced sputum improves the diagnosis of pulmonary tuberculosis in hospitalized patients in Gaborone, Botswana. Int J Tuberc Lung Dis 12: 1279–1285.

102. Soto A, Solari L, Agapito J, Acuna-Villaorduna C, Lambert M-L, et al. (2008) Development of a clinical scoring system for the diagnosis of smear-negative pulmonary tuberculosis. Braz J Infect Dis 12: 128–132.

103. Weber AM, Areerat P, Fischer JE, Thamthitiwat S, Olsen SJ, et al. (2008) Factors associated with diagnostic evaluation for tuberculosis among adults hospitalized for clinical pneumonia in Thailand. Infect Control Hosp Epidemiol 29: 648–657. doi:10.1086/588684

104. Chang KC, Leung CC, Yew WW, Tam CM (2008) Supervised and induced sputum among patients with smear-negative pulmonary tuberculosis. Eur Respir J 31: 1085–1090. doi:10.1183/09031936.00122907

105. Bell DJ, Dacombe R, Graham SM, Hicks A, Cohen D, et al. (2009) Simple measures are as effective as invasive techniques in the diagnosis of pulmonary tuberculosis in Malawi. Int J Tuberc Lung Dis 13: 99–104.

106. Scherer LC, Sperhacke RD, Ruffino-Netto A, Rossetti ML, Vater C, et al. (2009) Cost-effectiveness analysis of PCR for the rapid diagnosis of pulmonary tuberculosis. BMC Infect Dis 9: 216. doi:10.1186/1471-2334-9-216

107. Lin S-M, Ni Y-L, Kuo C-H, Lin T-Y, Wang T-Y, et al. (2010) Endobronchial ultrasound increases the diagnostic yields of polymerase chain reaction and smear for pulmonary tuberculosis. J Thorac Cardiovasc Surg 139: 1554–1560. doi:10.1016/j.jtcvs.2010.02.019

108. Kalawat U, Sharma KK, Reddy PNR, Kumar AG (2010) Study of bronchoalveolar lavage in clinically and radiologically suspected cases of pulmonary tuberculosis. Lung India 27: 122–124. doi:10.4103/0970-2113.68307

109. Oberhelman RA, Soto-Castellares G, Gilman RH, Caviedes L, Castillo ME, et al. (2010) Diagnostic approaches for paediatric tuberculosis by use of different specimen types, culture methods, and PCR: a prospective case-control study. Lancet Infect Dis 10: 612–620. doi:10.1016/S1473-3099(10)70141-9

110. Soto A, Solari L, Gotuzzo E, Acinelli R, Vargas D, et al. (2011) Performance of an algorithm based on WHO recommendations for the diagnosis of smear-negative pulmonary tuberculosis in patients without HIV infection. Trop Med Int Health 16: 424–430. doi:10.1111/j.1365-3156.2010.02715.x

111. Koole O, Thai S, Khun KE, Pe R, van Griensven J, et al. (2011) Evaluation of the 2007 WHO guideline to improve the diagnosis of tuberculosis in ambulatory HIV-positive adults. PLoS ONE 6: e18502. doi:10.1371/journal.pone.0018502

112. Soto A, Solari L, Díaz J, Mantilla A, Matthys F, et al. (2011) Validation of a clinical-radiographic score to assess the probability of pulmonary tuberculosis in suspect patients with negative sputum smears. PLoS ONE 6: e18486. doi:10.1371/journal.pone.0018486

113. Porskrog A, Bjerregaard-Andersen M, Oliveira I, Joaquím LC, Camara C, et al. (2011) Enhanced tuberculosis identification through 1-month follow-up of smear-negative tuberculosis suspects. Int J Tuberc Lung Dis 15: 459–464. doi:10.5588/ijtld.10.0353

114. Holtz TH, Kabera G, Mthiyane T, Zingoni T, Nadesan S, et al. (2011) Use of a WHO-recommended algorithm to reduce mortality in seriously ill patients with HIV infection and smear-negative pulmonary tuberculosis in South Africa: an observational cohort study. Lancet Infect Dis 11: 533–540. doi:10.1016/S1473-3099(11)70057-3

115. Walley J, Kunutsor S, Evans M, Thoulass J, Katabira E, et al. (2011) Validation in Uganda of the new WHO diagnostic algorithm for smear-negative pulmonary tuberculosis in HIV prevalent settings. J Acquir Immune Defic Syndr 57: e93–100. doi:10.1097/QAI.0b013e3182243a8c

116. Wilson D, Mbhele L, Badri M, Morroni C, Nachega J, et al. (2011) Evaluation of the World Health Organization algorithm for the diagnosis of HIV-associated sputum smear-negative tuberculosis. Int J Tuberc Lung Dis 15: 919–924. doi:10.5588/ijtld.10.0440

117. Alavi-Naini R, Cuevas LE, Squire SB, Mohammadi M, Davoudikia A-A (2012) Clinical and laboratory diagnosis of the patients with sputum smear-negative pulmonary tuberculosis. Arch Iran Med 15: 22–26. doi:012151/AIM.007

118. Mosimaneotsile B, Talbot EA, Moeti TL, Hone NM, Moalosi G, et al. (2003) Value of chest radiography in a tuberculosis prevention programme for HIV-infected people, Botswana. Lancet 362: 1551–1552.

119. Mohammed A, Ehrlich R, Wood R, Cilliers F, Maartens G (2004) Screening for tuberculosis in adults with advanced HIV infection prior to preventive therapy. Int J Tuberc Lung Dis 8: 792–795.

120. Sanchez A, Gerhardt G, Natal S, Capone D, Espinola A, et al. (2005) Prevalence of pulmonary tuberculosis and comparative evaluation of screening strategies in a Brazilian prison. Int J Tuberc Lung Dis 9: 633–639.

121. Day JH, Charalambous S, Fielding KL, Hayes RJ, Churchyard GJ, et al. (2006) Screening for tuberculosis prior to isoniazid preventive therapy among HIV-infected gold miners in South Africa. Int J Tuberc Lung Dis 10: 523–529.

122. Chheng P, Tamhane A, Natpratan C, Tan V, Lay V, et al. (2008) Pulmonary tuberculosis among patients visiting a voluntary confidential counseling and testing center, Cambodia. Int J Tuberc Lung Dis 12: 54–62.

123. Lewis JJ, Charalambous S, Day JH, Fielding KL, Grant AD, et al. (2009) HIV infection does not affect active case finding of tuberculosis in South African gold miners. Am J Respir Crit Care Med 180: 1271–1278. doi:10.1164/rccm.200806-846OC

124. Ayles H, Schaap A, Nota A, Sismanidis C, Tembwe R, et al. (2009) Prevalence of tuberculosis, HIV and respiratory symptoms in two Zambian communities: implications for tuberculosis control in the era of HIV. PLoS ONE 4: e5602. doi:10.1371/journal.pone.0005602

125. Monkongdee P, McCarthy KD, Cain KP, Tasaneeyapan T, Nguyen HD, et al. (2009) Yield of acid-fast smear and mycobacterial culture for tuberculosis diagnosis in people with human immunodeficiency virus. Am J Respir Crit Care Med 180: 903–908. doi:10.1164/rccm.200905-0692OC

126. Shah S, Demissie M, Lambert L, Ahmed J, Leulseged S, et al. (2009) Intensified tuberculosis case finding among HIV-Infected persons from a voluntary counseling and testing center in Addis Ababa, Ethiopia. J Acquir Immune Defic Syndr 50: 537–545. doi:10.1097/QAI.0b013e318196761c

127. Tamhane A, Chheng P, Dobbs T, Mak S, Sar B, et al. (2009) Predictors of smear-negative pulmonary tuberculosis in HIV-infected patients, Battambang, Cambodia. Int J Tuberc Lung Dis 13: 347–354.

128. Were W, Moore D, Ekwaru P, Mwima G, Bunnell R, et al. (2009) A simple screening tool for active tuberculosis in HIV-infected adults receiving antiretroviral treatment in Uganda. Int J Tuberc Lung Dis 13: 47–53.

129. Lawn SD, Edwards DJ, Kranzer K, Vogt M, Bekker L-G, et al. (2009) Urine lipoarabinomannan assay for tuberculosis screening before antiretroviral therapy diagnostic yield and association with immune reconstitution disease. AIDS 23: 1875–1880.

130. Agizew TB, Arwady MA, Yoon JC, Nyirenda S, Mosimaneotsile B, et al. (2010) Tuberculosis in asymptomatic HIV-infected adults with abnormal chest radiographs screened for tuberculosis prevention. Int J Tuberc Lung Dis 14: 45–51.

131. Cain KP, McCarthy KD, Heilig CM, Monkongdee P, Tasaneeyapan T, et al. (2010) An algorithm for tuberculosis screening and diagnosis in people with HIV. N Engl J Med 362: 707–716. doi:10.1056/NEJMoa0907488

132. Corbett EL, Zezai A, Cheung YB, Bandason T, Dauya E, et al. (2010) Provider-initiated symptom screening for tuberculosis in Zimbabwe: diagnostic value and the effect of HIV status. Bull World Health Organ 88: 13–21. doi:10.2471/BLT.08.055467

133. Bassett IV, Wang B, Chetty S, Giddy J, Losina E, et al. (2010) Intensive tuberculosis screening for HIV-infected patients starting antiretroviral therapy in Durban, South Africa. Clin Infect Dis 51: 823–829. doi:10.1086/656282

134. Churchyard GJ, Fielding KL, Lewis JJ, Chihota VN, Hanifa Y, et al. (2010) Symptom and chest radiographic screening for infectious tuberculosis prior to starting isoniazid preventive therapy: yield and proportion missed at screening. AIDS 24 Suppl 5: S19–27. doi:10.1097/01.aids.0000391018.72542.46

135. Nguyen DTM, Hung NQ, Giang LT, Dung NH, Lan NTN, et al. (2011) Improving the diagnosis of pulmonary tuberculosis in HIV-infected individuals in Ho Chi Minh City, Viet Nam. Int J Tuberc Lung Dis 15: 1528–1534, i. doi:10.5588/ijtld.10.0777

136. Rangaka MX, Gideon HP, Wilkinson KA, Pai M, Mwansa-Kambafwile J, et al. (2012) Interferon release does not add discriminatory value to smear-negative HIV-tuberculosis algorithms. Eur Respir J 39: 163–171. doi:10.1183/09031936.00058911

137. Suo J, Yu MC, Lee CN, Chiang CY, Lin TP (1996) Treatment of multidrug-resistant tuberculosis in Taiwan. Chemotherapy 42 Suppl 3: 20–23; discussion 30–33.

138. Maranetra KN (1996) Treatment of multidrug-resistant tuberculosis in Thailand. Chemotherapy 42 Suppl 3: 10–15; discussion 30–33.

139. Mangunnegoro H, Hudoyo A (1999) Efficacy of low-dose ofloxacin in the treatment of multidrug-resistant tuberculosis in Indonesia. Chemotherapy 45 Suppl 2: 19–25.

140. Maranetra_Chemother_1999.pdf (z.d.).

141. Yew WW, Chan CK, Chau CH, Tam CM, Leung CC, et al. (2000) Outcomes of patients with multidrug-resistant pulmonary tuberculosis treated with ofloxacin/levofloxacin-containing regimens. Chest 117: 744–751.

142. Kim HJ, Hong YP, Kim SJ, Lew WJ, Lee EG (2001) Ambulatory treatment of multidrug-resistant pulmonary tuberculosis patients at a chest clinic. Int J Tuberc Lung Dis 5: 1129–1136.

143. Furin JJ, Mitnick CD, Shin SS, Bayona J, Becerra MC, et al. (2001) Occurrence of serious adverse effects in patients receiving community-based therapy for multidrug-resistant tuberculosis. Int J Tuberc Lung Dis 5: 648–655.

144. Suárez PG, Floyd K, Portocarrero J, Alarcón E, Rapiti E, et al. (2002) Feasibility and cost-effectiveness of standardised second-line drug treatment for chronic tuberculosis patients: a national cohort study in Peru. Lancet 359: 1980–1989. doi:10.1016/S0140-6736(02)08830-X

145. Tupasi TE, Quelapio MID, Orillaza RB, Alcantara C, Mira NRC, et al. (2003) DOTS-Plus for multidrug-resistant tuberculosis in the Philippines: global assistance urgently needed. Tuberculosis (Edinb) 83: 52–58.

146. Mitnick C, Bayona J, Palacios E, Shin S, Furin J, et al. (2003) Community-based therapy for multidrug-resistant tuberculosis in Lima, Peru. N Engl J Med 348: 119–128. doi:10.1056/NEJMoa022928

147. Park SK, Lee WC, Lee DH, Mitnick CD, Han L, et al. (2004) Self-administered, standardized regimens for multidrug-resistant tuberculosis in South Korea. Int. J. Tuberc. Lung Dis 8: 361–368.

148. Van Deun A, Salim MAH, Das APK, Bastian I, Portaels F (2004) Results of a standardised regimen for multidrug-resistant tuberculosis in Bangladesh. Int J Tuberc Lung Dis 8: 560–567.

149. Palmero DJ, Ambroggi M, Brea A, De Lucas M, Fulgenzi A, et al. (2004) Treatment and follow-up of HIV-negative multidrug-resistant tuberculosis patients in an infectious diseases reference hospital, Buenos Aires, Argentina. Int J Tuberc Lung Dis 8: 778–784.

150. Choi SS, Jazayeri DG, Mitnick CD, Chalco K, Bayona J, et al. (2004) Implementation and initial evaluation of a Web-based nurse order entry system for multidrug-resistant tuberculosis patients in Peru. Stud Health Technol Inform 107: 202–206.

151. Vega P, Sweetland A, Acha J, Castillo H, Guerra D, et al. (2004) Psychiatric issues in the management of patients with multidrug-resistant tuberculosis. Int J Tuberc Lung Dis 8: 749–759.

152. Nathanson E, Gupta R, Huamani P, Leimane V, Pasechnikov AD, et al. (2004) Adverse events in the treatment of multidrug-resistant tuberculosis: results from the DOTS-Plus initiative. Int J Tuberc Lung Dis 8: 1382–1384.

153. Leimane V, Riekstina V, Holtz TH, Zarovska E, Skripconoka V, et al. (2005) Clinical outcome of individualised treatment of multidrug-resistant tuberculosis in Latvia: a retrospective cohort study. Lancet 365: 318–326. doi:10.1016/S0140-6736(05)17786-1

154. Ollé-Goig JE, Sandy R (2005) Outcomes of individualised treatment for multidrug-resistant tuberculosis before DOTS-plus. Int J Tuberc Lung Dis 9: 765–770.

155. Törün T, Güngör G, Ozmen I, Bölükbaşi Y, Maden E, et al. (2005) Side effects associated with the treatment of multidrug-resistant tuberculosis. Int J Tuberc Lung Dis 9: 1373–1377.

156. Chiang C-Y, Enarson DA, Yu M-C, Bai K-J, Huang R-M, et al. (2006) Outcome of pulmonary multidrug-resistant tuberculosis: a 6-yr follow-up study. Eur Respir J 28: 980–985. doi:10.1183/09031936.06.00125705

157. Shin SS, Pasechnikov AD, Gelmanova IY, Peremitin GG, Strelis AK, et al. (2006) Treatment outcomes in an integrated civilian and prison MDR-TB treatment program in Russia. Int J Tuberc Lung Dis 10: 402–408.

158. Tupasi TE, Gupta R, Quelapio MID, Orillaza RB, Mira NR, et al. (2006) Feasibility and cost-effectiveness of treating multidrug-resistant tuberculosis: a cohort study in the Philippines. PLoS Med 3: e352. doi:10.1371/journal.pmed.0030352

159. Nathanson E, Lambregts-van Weezenbeek C, Rich ML, Gupta R, Bayona J, et al. (2006) Multidrug-resistant tuberculosis management in resource-limited settings. Emerging Infect Dis 12: 1389–1397.

160. Holtz TH, Lancaster J, Laserson KF, Wells CD, Thorpe L, et al. (2006) Risk factors associated with default from multidrug-resistant tuberculosis treatment, South Africa, 1999-2001. Int J Tuberc Lung Dis 10: 649–655

161. Chalco K, Wu DY, Mestanza L, Muñoz M, Llaro K, et al. (2006) Nurses as providers of emotional support to patients with MDR-TB. Int Nurs Rev 53: 253–260. doi:10.1111/j.1466-7657.2006.00490.x

162. Leimane V, Leimans J (2006) Tuberculosis control in Latvia: integrated DOTS and DOTS-plus programmes. Euro Surveill 11: 29–33.

163. Prasad R, Verma SK, Sahai S, Kumar S, Jain A (2006) Efficacy and safety of kanamycin, ethionamide, PAS and cycloserine in multidrug-resistant pulmonary tuberculosis patients. Indian J Chest Dis Allied Sci 48: 183–186.

164. Cox HS, Kalon S, Allamuratova S, Sizaire V, Tigay ZN, et al. (2007) Multidrug-resistant tuberculosis treatment outcomes in Karakalpakstan, Uzbekistan: treatment complexity and XDR-TB among treatment failures. PLoS ONE 2: e1126. doi:10.1371/journal.pone.0001126

165. Törün T, Tahaoğlu K, Ozmen I, Sevim T, Ataç G, et al. (2007) The role of surgery and fluoroquinolones in the treatment of multidrug-resistant tuberculosis. Int J Tuberc Lung Dis 11: 979–985.

166. Shin S, Furin J, Bayona J, Mate K, Kim JY, et al. (2004) Community-based treatment of multidrug-resistant tuberculosis in Lima, Peru: 7 years of experience. Soc Sci Med 59: 1529–1539. doi:10.1016/j.socscimed.2004.01.027

167. Thomas A, Ramachandran R, Rehaman F, Jaggarajamma K, Santha T, et al. (2007) Management of multi drug resistance tuberculosis in the field: Tuberculosis Research Centre experience. Indian J Tuberc 54: 117–124.

168. Clark PM, Karagoz T, Apikoglu-Rabus S, Izzettin FV (2007) Effect of pharmacist-led patient education on adherence to tuberculosis treatment. Am J Health Syst Pharm 64: 497–505. doi:10.2146/ajhp050543

169. Acha J, Sweetland A, Guerra D, Chalco K, Castillo H, et al. (2007) Psychosocial support groups for patients with multidrug-resistant tuberculosis: five years of experience. Glob Public Health 2: 404–417. doi:10.1080/17441690701191610

170. Joshi J, Singh R, Gothi D (2007) Multidrug resistant tuberculosis: Role of previous treatment with second line therapy on treatment outcome. Lung India 24: 54. doi:10.4103/0970-2113.44211

171. Kwon YS, Kim YH, Suh GY, Chung MP, Kim H, et al. (2008) Treatment outcomes for HIV-uninfected patients with multidrug-resistant and extensively drug-resistant tuberculosis. Clin Infect Dis 47: 496–502. doi:10.1086/590005

172. Kim DH, Kim HJ, Park S-K, Kong S-J, Kim YS, et al. (2008) Treatment outcomes and long-term survival in patients with extensively drug-resistant tuberculosis. Am J Respir Crit Care Med 178: 1075–1082. doi:10.1164/rccm.200801-132OC

173. Shean KP, Willcox PA, Siwendu SN, Laserson KF, Gross L, et al. (2008) Treatment outcome and follow-up of multidrug-resistant tuberculosis patients, West Coast/Winelands, South Africa, 1992-2002. Int J Tuberc Lung Dis 12: 1182–1189.

174. Mitnick CD, Shin SS, Seung KJ, Rich ML, Atwood SS, et al. (2008) Comprehensive treatment of extensively drug-resistant tuberculosis. N Engl J Med 359: 563–574. doi:10.1056/NEJMoa0800106

175. Franke MF, Appleton SC, Bayona J, Arteaga F, Palacios E, et al. (2008) Risk factors and mortality associated with default from multidrug-resistant tuberculosis treatment. Clin Infect Dis 46: 1844–1851. doi:10.1086/588292

176. Karagöz T, Yazicioğlu Moçin O, Pazarli P, Senol T, Yetiş Duman D, et al. (2009) The treatment results of patients with multidrug resistant tuberculosis and factors affecting treatment outcome. Tuberk Toraks 57: 383–392.

177. Jeon DS, Kim DH, Kang HS, Hwang SH, Min JH, et al. (2009) Survival and predictors of outcomes in non-HIV-infected patients with extensively drug-resistant tuberculosis. Int J Tuberc Lung Dis 13: 594–600.

178. O’Donnell MR, Padayatchi N, Master I, Osburn G, Horsburgh CR (2009) Improved early results for patients with extensively drug-resistant tuberculosis and HIV in South Africa. Int J Tuberc Lung Dis 13: 855–861.

179. Seung KJ, Omatayo DB, Keshavjee S, Furin JJ, Farmer PE, et al. (2009) Early outcomes of MDR-TB treatment in a high HIV-prevalence setting in Southern Africa. PLoS ONE 4: e7186. doi:10.1371/journal.pone.0007186

180. Rao NA, Irfan M, Mahfooz Z (2009) Treatment outcome of multi-drug resistant tuberculosis in a tertiary care hospital in Karachi. J Pak Med Assoc 59: 694–698.

181. Malla P, Kanitz EE, Akhtar M, Falzon D, Feldmann K, et al. (2009) Ambulatory-based standardized therapy for multi-drug resistant tuberculosis: experience from Nepal, 2005-2006. PLoS ONE 4: e8313. doi:10.1371/journal.pone.0008313

182. Tabarsi P, Baghaei P, Jalali S, Farnia P, Chitsaz E, et al. (2009) Is standardized treatment appropriate for non-XDR multiple drug resistant tuberculosis cases? A clinical descriptive study. Scand J Infect Dis 41: 10–13. doi:10.1080/00365540802298079

183. Singla R, Sarin R, Khalid UK, Mathuria K, Singla N, et al. (2009) Seven-year DOTS-Plus pilot experience in India: results, constraints and issues. Int J Tuberc Lung Dis 13: 976–981.

184. Kliiman K, Altraja A (2009) Predictors of poor treatment outcome in multi- and extensively drug-resistant pulmonary TB. Eur Respir J 33: 1085–1094. doi:10.1183/09031936.00155708

185. Siqueira HR de, Freitas FAD de, Oliveira DN de, Barreto AMW, Dalcolmo MP, et al. (2009) Clinical evolution of a group of patients with multidrug-resistant TB treated at a referral center in the city of Rio de Janeiro, Brazil. J Bras Pneumol 35: 54–62.

186. Bloss E, Kuksa L, Holtz TH, Riekstina V, Skripconoka V, et al. (2010) Adverse events related to multidrug-resistant tuberculosis treatment, Latvia, 2000-2004. Int J Tuberc Lung Dis 14: 275–281.

187. Quelapio MID, Mira NRC, Orillaza-Chi RB, Belen V, Muñez N, et al. (2010) Responding to the multidrug-resistant tuberculosis crisis: mainstreaming programmatic management to the Philippine National Tuberculosis Programme. Int J Tuberc Lung Dis 14: 751–757.

188. Brust JCM, Gandhi NR, Carrara H, Osburn G, Padayatchi N (2010) High treatment failure and default rates for patients with multidrug-resistant tuberculosis in KwaZulu-Natal, South Africa, 2000-2003. Int J Tuberc Lung Dis 14: 413–419.

189. Heller T, Lessells RJ, Wallrauch CG, Bärnighausen T, Cooke GS, et al. (2010) Community-based treatment for multidrug-resistant tuberculosis in rural KwaZulu-Natal, South Africa. Int J Tuberc Lung Dis 14: 420–426.

190. Van Deun A, Maug AKJ, Salim MAH, Das PK, Sarker MR, et al. (2010) Short, highly effective, and inexpensive standardized treatment of multidrug-resistant tuberculosis. Am J Respir Crit Care Med 182: 684–692. doi:10.1164/rccm.201001-0077OC

191. Shin SS, Keshavjee S, Gelmanova IY, Atwood S, Franke MF, et al. (2010) Development of extensively drug-resistant tuberculosis during multidrug-resistant tuberculosis treatment. Am J Respir Crit Care Med 182: 426–432. doi:10.1164/rccm.200911-1768OC

192. Leimane V, Dravniece G, Riekstina V, Sture I, Kammerer S, et al. (2010) Treatment outcome of multidrug/extensively drug-resistant tuberculosis in Latvia, 2000-2004. Eur Respir J 36: 584–593. doi:10.1183/09031936.00003710

193. Kim DH, Kim HJ, Park S-K, Kong S-J, Kim YS, et al. (2010) Treatment outcomes and survival based on drug resistance patterns in multidrug-resistant tuberculosis. Am J Respir Crit Care Med 182: 113–119. doi:10.1164/rccm.200911-1656OC

194. Kvasnovsky CL, Cegielski JP, Erasmus R, Siwisa NO, Thomas K, et al. (2011) Extensively drug-resistant TB in Eastern Cape, South Africa: High Mortality in HIV negative and HIV positive patients. J Acquir Immune Defic Syndr. Available: http://www.ncbi.nlm.nih.gov/pubmed/21297482.

195. Jeon DS, Shin DO, Park SK, Seo JE, Seo HS, et al. (2011) Treatment outcome and mortality among patients with multidrug-resistant tuberculosis in tuberculosis hospitals of the public sector. J Korean Med Sci 26: 33–41. doi:10.3346/jkms.2011.26.1.33

196. Tang S, Zhang Q, Yu J, Liu Y, Sha W, et al. (2011) Extensively drug-resistant tuberculosis at a tuberculosis specialist hospital in Shanghai, China: clinical characteristics and treatment outcomes. Scand J Infect Dis 43: 280–285. doi:10.3109/00365548.2010.548080

197. Podewils LJ, Holtz T, Riekstina V, Skripconoka V, Zarovska E, et al. (2011) Impact of malnutrition on clinical presentation, clinical course, and mortality in MDR-TB patients. Epidemiol Infect 139: 113–120. doi:10.1017/S0950268810000907

198. Liu CH, Li L, Chen Z, Wang Q, Hu YL, et al. (2011) Characteristics and Treatment Outcomes of Patients with MDR and XDR Tuberculosis in a TB Referral Hospital in Beijing: A 13-Year Experience. PLoS ONE 6: e19399. doi:10.1371/journal.pone.0019399

199. Baghaei P, Tabarsi P, Dorriz D, Marjani M, Shamaei M, et al. (2011) Adverse effects of multidrug-resistant tuberculosis treatment with a standardized regimen: a report from Iran. Am J Ther 18: e29–34. doi:10.1097/MJT.0b013e3181c0806d

200. Lee J, Lee C-H, Kim DK, Yoon HI, Kim JY, et al. (2011) Retrospective comparison of levofloxacin and moxifloxacin on multidrug-resistant tuberculosis treatment outcomes. Korean J Intern Med 26: 153–159. doi:10.3904/kjim.2011.26.2.153

201. Bonnet M, Pardini M, Meacci F, Orrù G, Yesilkaya H, et al. (2011) Treatment of tuberculosis in a region with high drug resistance: outcomes, drug resistance amplification and re-infection. PLoS ONE 6: e23081. doi:10.1371/journal.pone.0023081

202. Chadha SS, Sharath BN, Reddy K, Jaju J, Vishnu PH, et al. (2011) Operational challenges in diagnosing multi-drug resistant TB and initiating treatment in Andhra Pradesh, India. PLoS ONE 6: e26659. doi:10.1371/journal.pone.0026659

203. Farley JE, Ram M, Pan W, Waldman S, Cassell GH, et al. (2011) Outcomes of multi-drug resistant tuberculosis (MDR-TB) among a cohort of South African patients with high HIV prevalence. PLoS ONE 6: e20436. doi:10.1371/journal.pone.0020436

204. Isaakidis P, Cox HS, Varghese B, Montaldo C, Da Silva E, et al. (2011) Ambulatory multi-drug resistant tuberculosis treatment outcomes in a cohort of HIV-infected patients in a slum setting in Mumbai, India. PLoS ONE 6: e28066. doi:10.1371/journal.pone.0028066

205. Kunawararak P, Pongpanich S, Chantawong S, Pokaew P, Traisathit P, et al. (2011) Tuberculosis treatment with mobile-phone medication reminders in northern Thailand. Southeast Asian J Trop Med Public Health 42: 1444–1451.

206. Alexy ER, Podewils LJ, Mitnick CD, Becerra MC, Laserson KF, et al. (2012) Concordance of programmatic and laboratory-based multidrug-resistant tuberculosis treatment outcomes in Peru. Int J Tuberc Lung Dis. 16: 364–369. doi:10.5588/ijtld.11.0511

207. Loveday M, Wallengren K, Voce A, Margot B, Reddy T, et al. (2012) Comparing early treatment outcomes of MDR-TB in decentralised and centralised settings in KwaZulu-Natal, South Africa. Int J Tuberc Lung Dis 16: 209–215. doi:10.5588/ijtld.11.0401

208. Palacios E, Franke M, Muñoz M, Hurtado R, Dallman R, et al. (2012) HIV-positive patients treated for multidrug-resistant tuberculosis: clinical outcomes in the HAART era. Int J Tuberc Lung Dis 16: 348–354. doi:10.5588/ijtld.11.0473
